# Optimizing single irrigation scheme to improve water use efficiency by manipulating winter wheat sink-source relationships in Northern China Plain

**DOI:** 10.1371/journal.pone.0193895

**Published:** 2018-03-08

**Authors:** Xuexin Xu, Yinghua Zhang, Jinpeng Li, Meng Zhang, Xiaonan Zhou, Shunli Zhou, Zhimin Wang

**Affiliations:** 1 College of Agronomy, China Agricultural University, Beijing, China; 2 Scientific Observation and Experiment Station of Wuqiao for Crops with High Water Use Efficiency, Ministry of Agriculture, Cangzhou, China; Institute of Genetics and Developmental Biology Chinese Academy of Sciences, CHINA

## Abstract

Improving winter wheat grain yield and water use efficiency (WUE) with minimum irrigation is very important for ensuring agricultural and ecological sustainability in the Northern China Plain (NCP). A three-year field experiment was conducted to determine how single irrigation can improve grain yield and WUE by manipulating the “sink-source” relationships. To achieve this, no-irrigation after sowing (W0) as a control, and five single irrigation treatments after sowing (75 mm of each irrigation) were established. They included irrigation at upstanding (W_U_), irrigation at jointing (W_J_), irrigation at booting (W_B_), irrigation at anthesis (W_A_) and irrigation at medium milk (W_M_). Results showed that compared with no-irrigation after sowing (W0), W_U_, W_J_, W_B_, W_A_ and W_M_ significantly improved mean grain yield by 14.1%, 19.9%, 17.9%, 11.6%, and 7.5%, respectively. W_J_ achieved the highest grain yield (8653.1 kg ha^-1^) and WUE (20.3 kg ha^-1^ mm^-1^), and W_B_ observed the same level of grain yield and WUE as W_J_. In comparison to W_U_, W_J_ and W_B_ coordinated pre- and post-anthesis water use while reducing pre-anthesis and total evapotranspiration (ET). They also retained higher soil water content above 180 cm soil layers at anthesis, increased post-anthesis water use, and ultimately increased WUE. W_J_ and W_B_ optimized population quantity and individual leaf size, delayed leaf senescence, extended grain-filling duration, improved post-anthesis biomass and biomass remobilization (source supply capacity) as well as post-anthesis biomass per unit anthesis leaf area (P_ost_BA-leaf ratio). W_J_ also optimized the allocation of assimilation, increased the spike partitioning index (SPI, spike biomass/biomass at anthesis) and grain production efficiency (GPE, the ratio of grain number to biomass at anthesis), thus improved mean sink capacity by 28.1%, 5.7%, 21.9%, and 26.7% in comparison to W0, W_U_, W_A_ and W_M_, respectively. Compared with W_A_ and W_M_, W_J_ and W_B_ also increased sink capacity, post-anthesis biomass and biomass remobilization. These results demonstrated that single irrigation at jointing or booting could improve grain yield and WUE via coordinating the “source-sink” relationships with the high sink capacity and source supply capacity. Therefore, we propose that under adequate soil moisture conditions before sowing, single irrigation scheme from jointing to booting with 75 mm irrigation amount is the optimal minimum irrigation practice for wheat production in this region.

## Introduction

As the main winter wheat growing region in China, the Northern China Plain (NCP) provides more than 60% of the nation’s wheat production [1]. Because rainfall does not occur in synchronization with wheat growth stages, the natural precipitation is insufficient in the region and irrigation is required [2]. A supplementary irrigation of three or four times with more than 300 mm water was applied to achieve a high wheat yield [3]. As a result, over-exploitation of ground water threatened sustainable agricultural development and water use efficiency (WUE) was significantly reduced [4–5]. This agro-environmental challenge makes understanding the theory and technology to improve WUE and ensure food security in the NCP vital.

Limited irrigation, reducing irrigation times and irrigation amount, could be considered for saving water and improving WUE in the NCP [6]. It can induce soil water deficit at noncritical growth stages and ensure water supply at critical growth stages of wheat [2]. Previous studies have shown that irrigation frequency can be reduced to two irrigation events (at jointing and anthesis) reducing water consumption, improving grain yield and WUE [4, 7–8]. However, single irrigation scheme might be another strategy to save water, increase grain yield and WUE due to the decline of available water resources in NCP [7, 9–10].

Grain yield and WUE are also affected by individual and population traits, and “sink-source” relationships [8, 11]. Optimizing “sink-source” relationships could increase biomass and grain yield [12–16]. Many studies have explored theories and means to achieve high yield by optimizing “sink-source” relationships [12, 16]. However, many of these studies focused on the “sink-source” relationships based on individual grain weight of the individual plant [15–17], and the effects of population “sink-source” relationships based on final grain yield require further exploration. Many factors affect the “sink-source” relationships, including genotype, air temperature, rainfall and irrigation at different growth phases. However, irrigation is one of the most important factors affecting grain yield and WUE by manipulating “sink-source” relationships directly or indirectly [16, 18–20]. In areas where groundwater is seriously over-exploited in NCP, water shortages are becoming more serious [21], and irrigation is allowed only once during the wheat growth period. Under single irrigation conditions, optimizing irrigation timing to achieve the highest grain yield and WUE is vital. In our opinion, water distribution and the coordination of the “sink-source” relationships must be synthetically considered for optimizing the timing of single irrigation applications.

The objectives of this study were: (i) to determine the best irrigation timing in order to obtain high grain yield and improve WUE; (ii) to explore the mechanism of high grain yield and WUE under optimal single irrigation time based on the sink and source traits and the “sink-source” relationships at field level.

## Materials and methods

### Ethics statement

Wuqiao Experimental Station of China Agricultural University is a department of China Agricultural University. The farming operations of this experiment were similar to the rural farmers’ operations and did not involve endangered or protected species; no specific permissions were required in the experimental site; the operations were approved by College of Agronomy, China Agricultural University.

### Field descriptions

**Fig 1:**
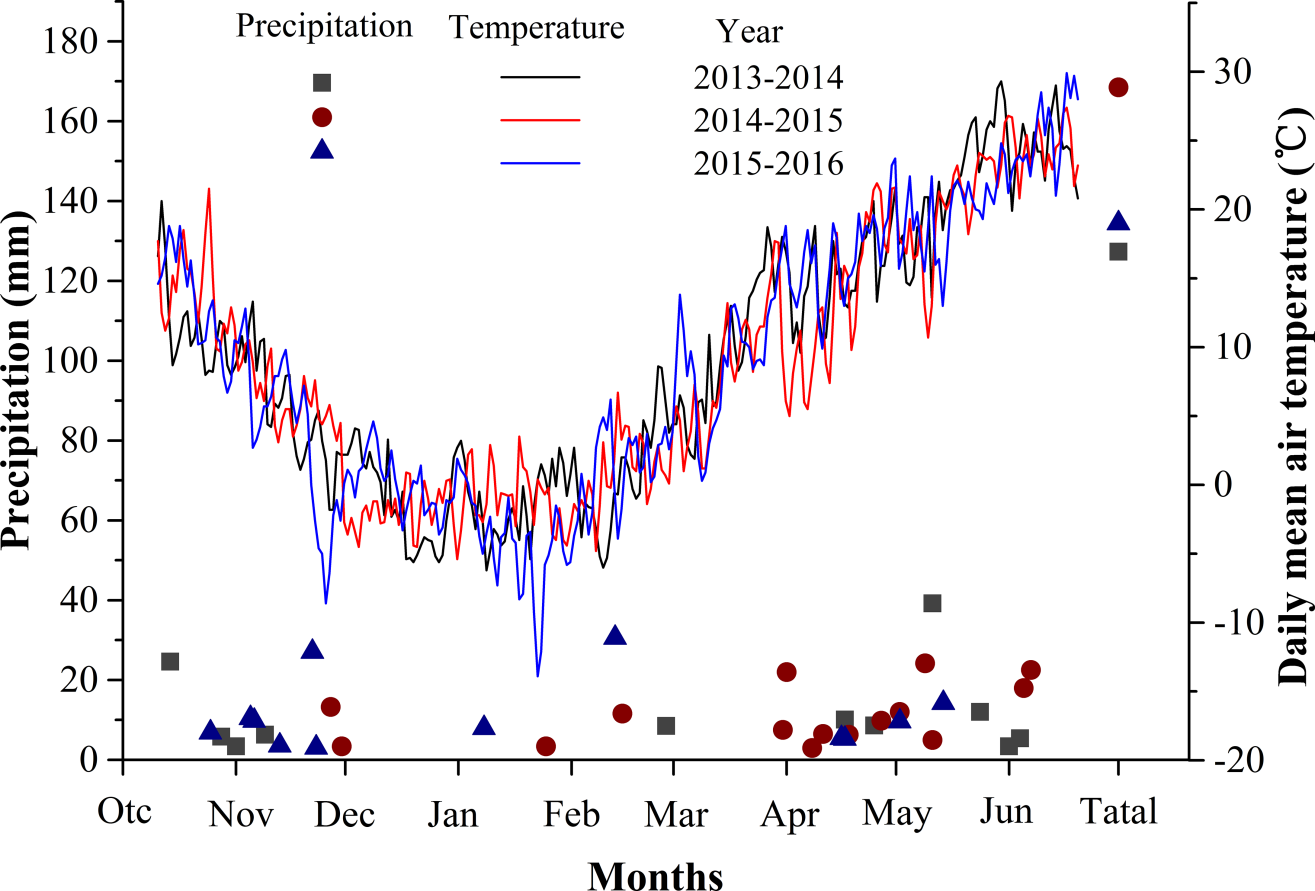
Precipitation and daily mean air temperature during 2013–2014, 2014–2015 and 2015–2016 growing seasons in WuQiao, Hebei Province.

The experiment was carried out during the 2013–2014, 2014–2015 and 2015–2016 growing seasons under field conditions at Wuqiao Experimental Station of China Agricultural University at Cangzhou (37°41′N, 116°36′ E), Hebei Province, China. Field soil type was determined to be clay-loam soil. Soil bulk density and field capacity were measured in 0–200 cm soil depth (20 cm increment) and are presented in Table 1. The organic matter, total nitrogen, hydrolysable nitrogen, available phosphorus and available potassium in the topsoil (0–20 cm) of the experimental plots were 12.1 g kg^-1^, 1.1 g kg^-1^, 80.6 mg kg^-1^, 45.3 mg kg^-1^ and 122.2 mg kg^-1^, respectively. Precipitation and daily mean air temperature in the 2013–2014, 2014–2015 and 2015–2016 growing seasons are shown in Fig 1.

**Table 1:** Soil bulk density and field capacity at 0–200 cm soil depth with 20 cm increment.

Soil layer (cm)	0–20	20–40	40–60	60–80	80–100	100–120	120–140	140–160	160–180	180–200
Bulk density (g cm^-3^)	1.45	1.48	1.48	1.48	1.49	1.48	1.49	1.51	1.50	1.51
Field capacity (%)	29.29	26.98	26.56	26.26	26.61	26.51	26.84	26.04	26.23	26.45

### Experimental design

Supplemental irrigation was administered according to the reported irrigation method [22] before sowing, the target relative soil water content of 0–200 cm soil layers was 80% of field capacity, and soil water content was irrigated to 81.3%, 80.0% and 81.6% of field capacity in the 2013–2014, 2014–2015 and 2015–2016 growing seasons before sowing, respectively. Crop developmental stages were categorized using the Zadoks scale [23]. No irrigation after sowing as a control (W0), five single irrigation treatments after sowing (75 mm of each irrigation) were established as the following: irrigation at Z30 (upstanding, W_U_), irrigation at Z31 (jointing, W_J_), irrigation at Z45 (booting, W_B_), irrigation at Z61 (anthesis, W_A_) and irrigation at Z75 (medium milk, W_M_). Water was irrigated evenly to the plots through surface irrigation with a 4-inch plastic-coated hose, and a flow meter was installed near the outlet of the hose to record the water used. Each experimental plot was 8 m × 5 m with rows spaced 0.16 m apart, and the experimental design was a randomized complete block design with three replications. A non-irrigated zone of 1 m wide was maintained to minimize the effects of adjacent plots.

### Crop management

The straw stubble of the preceding maize crop was plowed into the cropland before fertilizer was applied. A total of 180 kg N ha^-1^ (as urea), 140 kg P_2_O_5_ ha^-1^ (as diammonium phosphate), 75 kg K_2_O ha^-1^ (as potassium chloride) and 15 kg Zn ha^-1^ (as zinc sulfate) were broadcasted and incorporated into the upper 20 cm soil layer by rotary tillage prior to sowing, and no fertilizer was applied during growth. The high-yielding winter wheat cultivar “Jimai 22” (*Triticum aestivum L*.) was used in all the experiments. It was sown annually on 13 October 2013, 14 October 2014 and 12 October 2015. Plant density after emergence was 525 plants m^-2^. Additional protective measures were taken to assure the healthy growth of the wheat crop, such as the spraying of herbicides at the re-greening period, and the application of insecticides before anthesis. No significant incidence of pests, diseases or weeds was observed in any of the treatment sites during the experiment.

### Data acquisition and analysis

#### Crop phenology

Crop phenology was recorded using the Zadoks scale [23], following the average phenology of the plot (when 50% of shoots reached at main developmental stage). The corresponding dates were recorded when 50% of spikes extruded at least one anther (beginning of anthesis, Z61) and the grain was difficult to divide by the thumbnail (maturity, Z91). Days to anthesis (DTA) and days to maturity (DTM) were calculated as days after sowing to anthesis and days after sowing to maturity, respectively; Grain-filling duration (GFD) was calculated as the difference between DTA and DTM.

#### Estimating crop evapotranspiration

Soil samples were collected from 0.2 m increments to a depth of 2 m by using a soil corer in all experimental plots. Measurements were performed at the sowing (Z00), jointing (Z31), beginning of anthesis (Z61), medium milk (Z75) and maturity (Z91) stages. The soil water content was determined using the oven-drying method [24]. Crop evapotranspiration (ET) during the growth stage was calculated according to water balance equation [4] as below:

ET=I+P−R−D±SW

Where *ET* (mm) is crop evapotranspiration; *I* (mm) and *P* (mm) is irrigation and precipitation, respectively; *R* (mm) is surface runoff (based on the presence of beds around the plots and thus assuming that surface runoff was not significant); *D* (mm) is the downward flux below the crop root zone. Soil water measurements did not account for deep percolation, indicating negligible drainage at the site; *SW* (mm) represents the change in stored soil water (0–200 cm) between two specific stages of the soil profile exploited by root.

The ratio of seasonal crop evapotranspiration to total crop evapotranspiration was calculated by using the following equation [25]:

$$R=\frac{ETs}{ET}\times 100\%$$

Where *R* represents the ratio of seasonal crop evapotranspiration to total crop evapotranspiration; *ETs* represents seasonal crop evapotranspiration; *ET* is the total crop evapotranspiration throughout the winter wheat growing season.

#### Aboveground biomass and leaf size

Two 1 m inner rows of plants from each plot were cut at ground level at anthesis (Z61) and maturity (Z91) stages. These plants were separated into stem + sheath, top three leaves, remaining green leaves, withered leaves, spike axis + glume and grains (only at maturity). The green plant organs were oven baked for 30 min at 105°C to deactivate the enzymes, and subsequently all plant samples were oven-dried at 75°C until they were a constant weight to determine aboveground biomass. The post-anthesis biomass and biomass remobilization during grain filling was calculated using the method developed by Chu et al. [22], as follows:

$$Post‑anthesis\;biomass\;(kg\;m^{‑2})=biomass\;at\;maturity–biomass\;at\;anthesis.$$

$$Biomass\;remobilization\;(kg\;m^{‑2})=biomass\;at\;anthesis–biomass\;at\;maturity\;without\;grain.$$

At anthesis stage, the area of top three leaves and remaining green leaves were measured using a LI-3100 area meter (Li-Cor, Inc., Lincoln, Nebraska, USA), and green leaf area index (LAI) was calculated; Twenty plants were randomly chosen for calculating the leaf area of a single plant at anthesis, the leaf area was calculated using the following equation [26]:

Leafarea=leaflength×leafwidth×0.78

#### Chlorophyll content

The chlorophyll content of the flag, second and third leaves from top were measured with a SPAD-502 Minolta chlorophyll meter (Spectrum Technologies, Plainfield, IL, USA). These measurements were undertaken in ten leaves per plot at 6-day intervals starting 6 days after anthesis (6 DAA) until 30 DAA.

#### Grain yield and WUE

Grain yield (with 13% water content) was measured from an area of 4 m^2^ in each plot at maturity. The number of spikes, the number of grains per spike and 1,000-grain weight (with 13% water content) was also investigated at harvest.

WUE was defined as follows [3]:

$$WUE=\frac{Y}{ET}$$

Where *WUE* (kg ha^-1^ mm^-1^) is the water use efficiency for grain yield; *Y* (kg ha^-1^) is the grain yield at maturity; *ET* (mm) is the total crop evapotranspiration over the growing season of winter wheat.

#### Sink and source indicators

Grain number per unit area (sink capacity), post-anthesis biomass per unit anthesis leaf area (P_ost_BA-leaf ratio), grain production efficiency (GPE, the ratio of grain number to biomass at anthesis) [27], spike partitioning index (SPI, spike biomass/biomass at anthesis) [28] and harvest index (HI, grain weight/ biomass at maturity) were calculated.

### Statistical analysis

Analyses of variance (ANOVA) were performed using the general linear model procedure in the SPSS 17.0 (SPSS Inc., Chicago, IL, USA); the combined ANOVA was also carried out across years, irrigations and their interactions. Treatment means were compared each year using the least significant difference test (*P* = 0.05). Figures were created using OriginPro 2016 (OriginLab Corporation, Northampton, MA, USA) and Microsoft 2003 (Microsoft, Redmond, WA, USA); bars in figures represent the standard errors.

## Results

Combined analysis of variance shown that year had a significant effect on the remaining traits, except for grain number per spike (Table 2); all the 23 traits were determined mainly by irrigation (*P* < 0.001); while days to anthesis, grain-filling duration, biomass at anthesis and maturity, post-anthesis biomass, 1,000-grain weight, WUE, and P_ost_BA-leaf ratio were influenced significantly by year × irrigation (Y × Irr) interaction.

**Table 2:** Mean squares from the combined analysis of variance for wheat phenology, evapotranspiration (ET), source and sink traits, source-sink relationships, grain yield and water use efficiency during the 2013–2016 growing seasons.

Traits	Source of variation			
	Year (Y)	Irrigation (Irr)	Y×Irr	Error
Degrees of freedom	2	5	10	36
Day to anthesis	57.1 *** ^3^	17.7 ***	0.6 **	0.2
Day to maturity	78.7 ***	17.0 ***	0.3 ^n.s.^	0.2
Grain-filling duration	13.6 ***	25.6 ***	0.80 *	0.4
ET (Z00 ^1^-Z31)	1347.4 ***	852.9 ***	3.2 ^n.s.^	19.8
ET (Z31-Z61)	1147.2 ***	989.3 ***	10.6 ^n.s.^	46.2
ET (Z61-Z91)	215.9 *	1212.9 ***	8.2 ^n.s.^	44.5
ET total	1558.6 ***	3195.4 ***	22.1 ^n.s.^	69.4
LAI ^2^ of top three leaves	0.2 ***	6.7 ***	0.006 ^n.s.^	0.01
LAI of total green leaves	0.7 ***	11.4 ***	0.03 ^n.s.^	0.02
Biomass at anthesis	0.01 ***	0.07 ***	3.5 10^−4 **^	1.2 10^−4^
Post-anthesis biomass	0.02 ***	0.01 ***	2.7 10^−4 **^	8.2 10^−5^
Biomass remobilization	3.3 10^−3 ***^	2.8 10^−3 ***^	6.4 10^−5 n.s.^	6.8 10^−5^
Biomass at maturity	0.06 ***	0.1 ***	8.9 10^−4 ***^	1.2 10^−4^
Sink capacity	1.7 **	39.9 ***	0.09 ^n.s.^	0.2
Spike number	1307.9 ***	17940.4 ***	182.3 ^n.s.^	101.7
Grain number per spike	0.5 ^n.s.^	28.6 ***	0.2 ^n.s.^	0.2
1000-grain weight	164.6 ***	29.7 ***	0.6 ^n.s.^	0.4
Grain yield	3770317.3 ***	2493840.0 ***	60023.8 ^n.s.^	35697.0
Harvest index	1.7 10^−3 ***^	1.6 10^−3 ***^	2.4 10^−5 n.s.^	1.9 10^−5^
Water use efficiency	41.2 ***	4.5 ***	0.4 *	0.2
P_ost_BA-leaf ratio	3886.1 ***	6130.7 ***	100.4 *	40.4
Grain production efficiency	9.7 ***	4.4 ***	0.09 ^n.s.^	0.1
Spike partitioning index	6.5 10^−5 ***^	2.7 10^−4 ***^	7.7 10^−6 n.s.^	5.0 10^−6^

^1^ Z00, Zadoks stage 00 (dry seed); Z31, first node is detectable; Z61, beginning of anthesis; Z91, caryopsis hard.

^2^ LAI, leaf area index; P_ost_BA-leaf ratio, post-anthesis biomass per unit anthesis leaf area.

^3 n.s.^,*, ** and *** mean no significant difference at *P* = 0.05, difference at *P* < 0.05, *P* < 0.01 and *P* < 0.001, respectively.

### Wheat phenology

As shown in Table 3, days to maturity (DTM) in W0 were significantly lower than in irrigation treatments; no significant difference was observed in DTM among irrigation treatments throughout the three-year experiment. Compared with W0, W_U_ and W_J_ extended the days to anthesis (DTA) by 3–4 d and 1–2 d, respectively; there was no significant difference in DTA among W0, W_B_, W_A_ and W_M_ in the 2013–2016 growing seasons. W_J_, W_B_, W_A_ and W_M_ extended the grain-filling duration (GFD) by 1–3 d, 2–4 d, 3–5 d and 3–5 d in comparison to W0, respectively; no significant difference was observed between W0 and W_U_ throughout the three-year experiment. These results showed that single irrigation at jointing (W_J_) could increase DTA and GFD, simultaneously, in comparison to W0.

**Table 3:** Days during different growing periods of winter wheat in the 2013–2014, 2014–2015 and 2015–2016 growing seasons.

Treatment	Days (d)								
	2013–2014			2014–2015			2015–2016		
	DTA^1^	DTM	GFD	DTA	DTM	GFD	DTA	DTM	GFD
W_U_	204a ^2^	238a	34c	208a	242a	34b	208a	240a	32c
W_J_	203ab	238a	35bc	205b	242a	37a	205b	240a	35b
W_B_	202bc	238a	36ab	204c	242a	38a	204c	240a	36ab
W_A_	201c	238a	37a	204c	242a	38a	204c	241a	37a
W_M_	201c	238a	37a	204c	242a	38a	204c	241a	37a
W0	201c	235b	34c	204c	239b	35b	204c	236b	32c

^1^ DTA, days to anthesis; DTM, days to maturity; GFD, grain-filling duration.

^2^ Mean values within columns followed by the different letters are statistically significant at *P* < 0.05 level.

### Crop evapotranspiration (ET)

The total ET and post-anthesis seasonal ET of W0 were significantly lower than those of irrigation treatments; seasonal ET of W0 from Z00 to Z61 was lower than that of W_U_ and W_J_ in three-year experiments (Table 4). Under single irrigation conditions, compared with W_U_, the mean total ET of W_J_, W_B_, W_A_ and W_M_ was lower by 3.4%, 4.4%, 5.9% and 7.3%, respectively. Seasonal ET of W_U_ from Z00 to Z61 was significantly higher than that of the rest of the irrigation treatments. During Z61 to Z91, the highest seasonal ET and evapotranspiration ratio were observed in W_A_ and there were no significant differences among W_J_, W_B_, W_A_ and W_M_ (Table 4); the post-anthesis seasonal ET and evapotranspiration ratio of W_U_ were lower in comparison to the rest of the irrigation treatments.

**Table 4:** Crop evapotranspiration (ET) in different growth periods in the 2013–2014, 2014–2015 and 2015–2016 growing seasons.

Treatments	Z00 ^1^ to Z31		Z31 to Z61		Z61 to Z91		Z00 to Z91
	ETs ^2^	Ratio	ETs	Ratio	ETs	Ratio	ET
	(mm)	(%)	(mm)	(%)	(mm)	(%)	(mm)
2013–2014							
W_U_	153.3a ^3^	35.6a	144.9a	33.6a	132.6b	30.8e	430.8a
W_J_	132.1b	31.4c	141.5ab	33.6a	147.6a	35.0c	421.3ab
W_B_	132.1b	32.0c	129.7bc	31.4abc	151.0a	36.6b	412.8bc
W_A_	132.1b	32.7bc	118.6c	29.3c	153.6a	38.0a	404.3c
W_M_	132.1b	33.0b	118.6c	29.7bc	149.1a	37.3ab	399.8c
W0	132.1b	35.6a	118.6c	31.9ab	120.7c	32.5d	371.4d
2014–2015							
W_U_	146.9a	33.2a	157.2a	35.5a	138.3bc	31.3e	442.3a
W_J_	122.7b	28.7d	154.6a	36.1a	150.4ab	35.2cd	427.7ab
W_B_	122.7b	28.9d	147.3ab	34.7ab	154.8a	36.4bc	424.8bc
W_A_	122.7b	29.3cd	135.4b	32.3c	160.8a	38.4a	418.9bc
W_M_	122.7b	29.9c	135.4b	33.0bc	152.2a	37.1ab	410.4c
W0	122.7b	31.5b	135.4b	34.8ab	131.2c	33.7d	389.4d
2015–2016							
W_U_	165.9a	36.7a	154.0a	34.1a	132.0b	29.2d	452.0a
W_J_	139.7b	32.4d	147.1ab	34.1a	144.7a	33.5b	431.5b
W_B_	139.7b	32.6cd	139.0bc	32.4bc	150.3a	35.0ab	428.7b
W_A_	139.7b	33.0cd	132.1c	31.2d	152.2a	35.9a	423.9b
W_M_	139.7b	33.4c	132.1c	31.6cd	146.1a	35.0ab	417.8b
W0	139.7b	35.3b	132.1c	33.3ab	124.3b	31.4c	396.1c

^1^ Z00, Zadoks stage 00 (dry seed); Z31, first node is detectable; Z61, beginning of anthesis; Z91, caryopsis hard.

^2^ ETs, seasonal crop evapotranspiration; ET, total crop evapotranspiration.

^3^ Mean values within columns followed by the different letters are statistically significant at *P* < 0.05 level.

**Fig 2:**
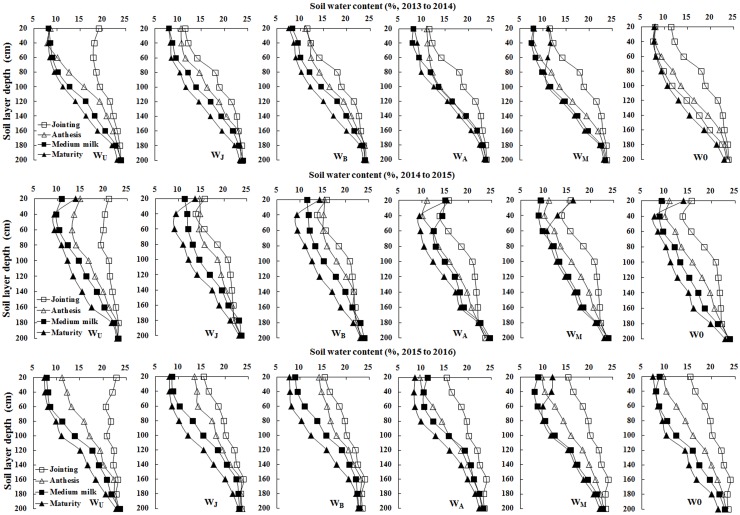
Soil water moisture under six treatments at jointing, anthesis, medium milk and maturity stages in the 2013–2014, 2014–2015 and 2015–2016 growing seasons.

Soil water consumption above the 100 cm soil layers in W_U_ was higher than in the other treatments during jointing (Z31) and anthesis (Z61) stage (Fig 2). The soil water content of W_U_ above the 120 cm soil layers at anthesis and in the 40 to 180 cm soil layers at medium milk was significantly lower than those of W_J_ and W_B_ (Fig 2). After the medium milk stage (Z75), there was little available soil water in W_U_ from the 0 to 80 cm soil layers; compared with W_U_, W_J_ and W_B_ increased soil water consumption from the 40 to 180 cm and 0 to 160 cm soil layers, respectively, in the 2013–2014 growing season, from the 40 to 180 cm soil layers in the 2014–2015 growing season and from 60 to 180 cm and 0 to 140 cm soil layers, respectively, in the 2015–2016 growing season; single irrigation at the anthesis and medium milk stages decreased the soil water consumption below the 120 cm and 60 cm soil layers than other treatments, respectively. These results indicated that W_J_ and W_B_ could coordinate pre- and post-anthesis water consumption by decreasing pre-anthesis water consumption and increasing post-anthesis water consumption.

### Grain yield and WUE

The spike number, grain number per spike and grain yield of irrigation treatments were significantly higher than they were in W0 during the 2013–2016 growing seasons (Table 5); compared with W0, W_U_, W_J_ and W_B_ increased the mean spike number by 18.9%, 11.4% and 7.6%, respectively; whereas no significant difference was observed among W0, W_A_ and W_M_. Grain number per spike, grain yield and WUE in W_J_ were the highest in three growing seasons. The grain number per spike in W_B_ was significantly lower than W_J_, but it was higher than in W_U_, W_A_ and W_M_ over the three-year environment. Compared with W_U_, W_A_, and W_M_, the mean grain yield of W_J_ was higher by 5.0%, 7.4% and 11.5%, respectively, while the mean WUE of W_J_ was higher by 8.6%, 4.5% and 6.7%, respectively; no significant difference was observed in grain yield and WUE between W_J_ and W_B_. The 1,000-grain weight in W_J_ was significantly lower than in W0, W_B_, W_A_ and W_M_, whereas no significant differences were observed between W_U_ and W_J_. These findings indicated that single irrigation at jointing and booting could improve grain yield and WUE effectively.

**Table 5:** Grain yield, yield components and water use efficiency (WUE) under six irrigation treatments in the 2013–2014, 2014–2015 and 2015–2016 growing seasons.

Treatments	SN^1^	GNPS	TGW	GY	WUE
	(10^4^ spike ha^-1^)	(grain spike^-1^)	(g)	(kg ha^-1^)	(kg ha^-1^ mm^-1^)
2013–2014					
W_U_	706.9a ^2^	30.6c	48.5c	8833.5ab	20.5c
W_J_	655.6b	34.9a	47.6c	9187.7a	21.8a
W_B_	633.3c	32.8b	51.4b	8908.5ab	21.6a
W_A_	604.9d	30.7c	52.9a	8624.1bc	21.3ab
W_M_	603.5d	30.1cd	52.6a	8399.1c	21.0abc
W0	600.0d	29.8d	51.2b	7648.5d	20.6bc
2014 to 2015					
W_U_	709.7a	31.0c	43.1c	7830.3b	17.7c
W_J_	672.9b	34.7a	42.8c	8217.7a	19.2a
W_B_	645.1c	33.3b	45.8ab	8199.0a	19.3a
W_A_	618.1d	31.3c	46.6a	7739.7b	18.5ab
W_M_	608.3d	30.3d	46.3a	7503.0bc	18.3bc
W0	606.9d	30.2d	44.7b	7129.8c	18.3bc
2015 to 2016					
W_U_	705.6a	30.7cd	44.7c	8052.6b	17.8bc
W_J_	660.4b	34.2a	44.3c	8553.9a	19.8a
W_B_	642.4c	33.1b	46.7b	8438.3a	19.7a
W_A_	592.4d	31.4c	48.4a	7800.3b	18.4b
W_M_	580.6d	30.7cd	47.8a	7380.4c	17.7c
W0	577.8d	30.5d	45.7b	6880.8d	17.4c

^1^ SN, Spike number; GNPS, Grain number per spike; TGW, 1,000-grain weight; GY, Grain yield

^2^ Mean values within columns followed by the different letters are statistically significant at *P* < 0.05 level.

### Source characteristics

#### Leaf size and LAI of wheat population

**Fig 3:**
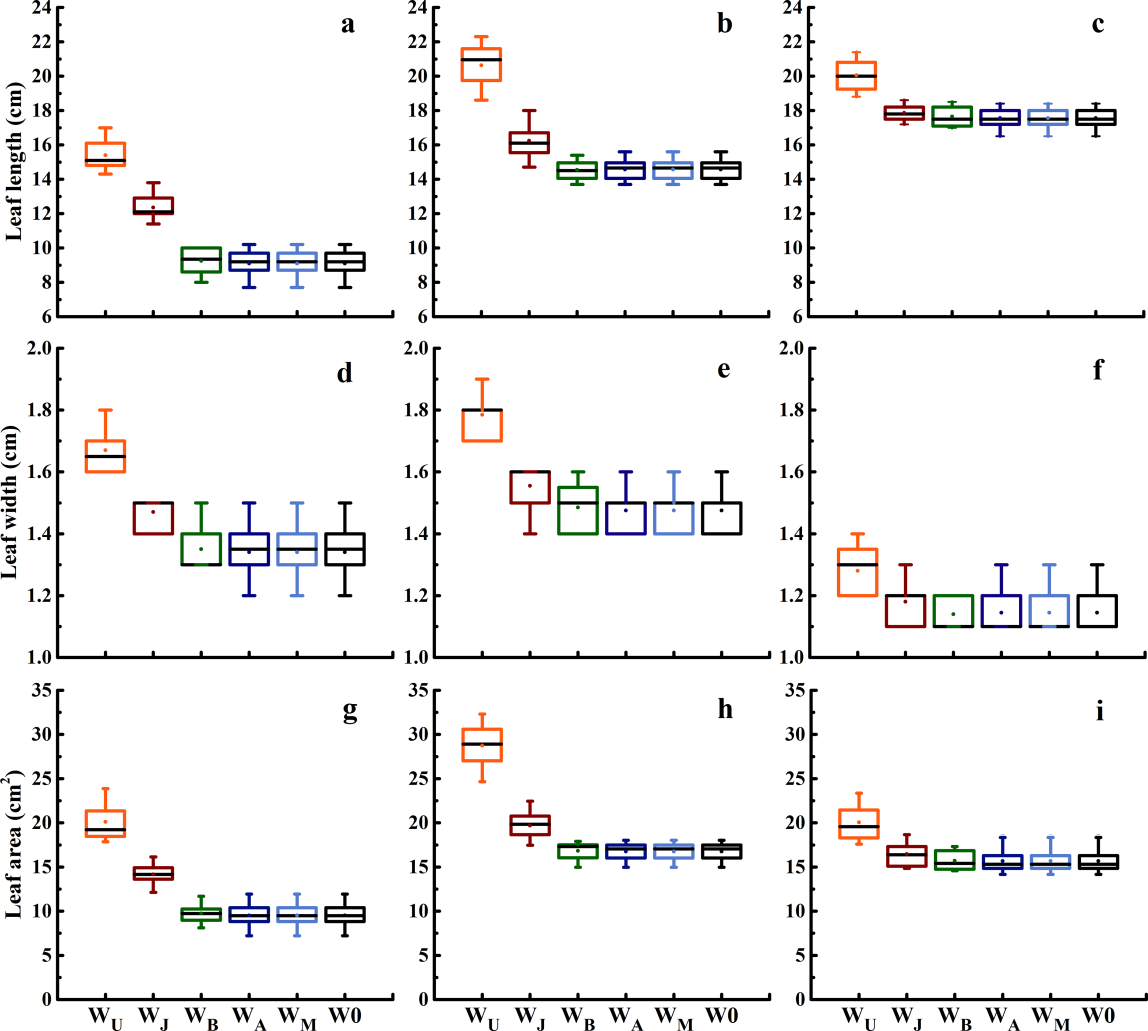
**Leaf length (top), width (middle) and area (bottom) of the flag (a, d), second (b, e) and third leaf (c, f) at anthesis under six treatments in the 2015–2016 growing season.** Box boundaries indicate upper and lower quartiles, whisker caps indicate maximum and minimum value, black solid horizontal lines indicate medians and solid dots indicate mean value.

W_U_ was observed the highest length, width and area of the flag, second and third leaves (Fig 3); the length, width and area of the flag and second leaf in W_J_ were higher than in W0, W_B_, W_A_, and W_M_, whereas no significant difference was observed in the leaf length, width and area of the third leaf among W0, W_J_, W_B_, W_A_, and W_M_.

**Fig 4:**
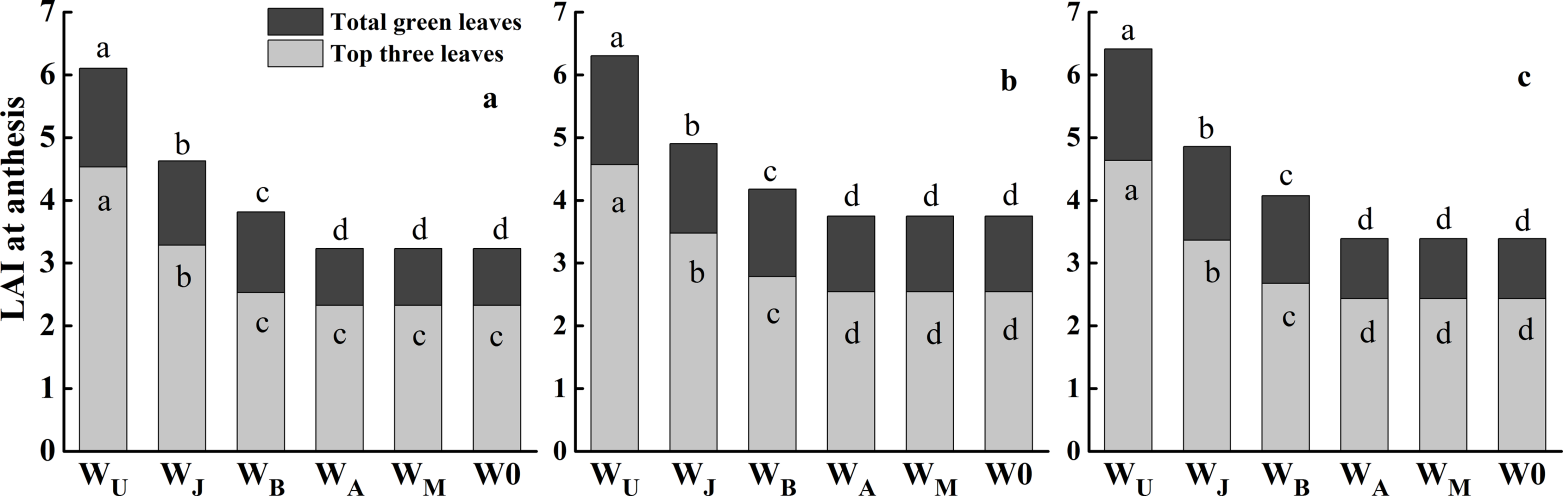
**Leaf area index (LAI) of top three leaves and total green leaves at anthesis under six treatments in the 2013–2014 (a), 2014–2015 (b) and 2015–2016 (c) growing seasons.** Different letters in the figure indicate statistical differences among treatments (LSD_*P*<0.05_).

The LAI of top three and total green leaves at anthesis were shown in Fig 4. The variations of LAI were consistent across three growing seasons. W_U_ showed the highest LAI of the top three leaves (as high as 4.6) and total green leaves (as high as 6.3), followed by W_J_, W_B_, W_A_, W_M_ and W0 over three growing seasons, while there was no significant difference in LAI of the top three leaves among W_B_, W_A_, W_M_ and W0 in the 2013–2014 growing season. These results indicated that leaf size and LAI were related to the timing of irrigation application, and they were decreased if irrigation was delayed from upstanding to anthesis stage.

#### Chlorophyll content (SPAD)

**Fig 5:**
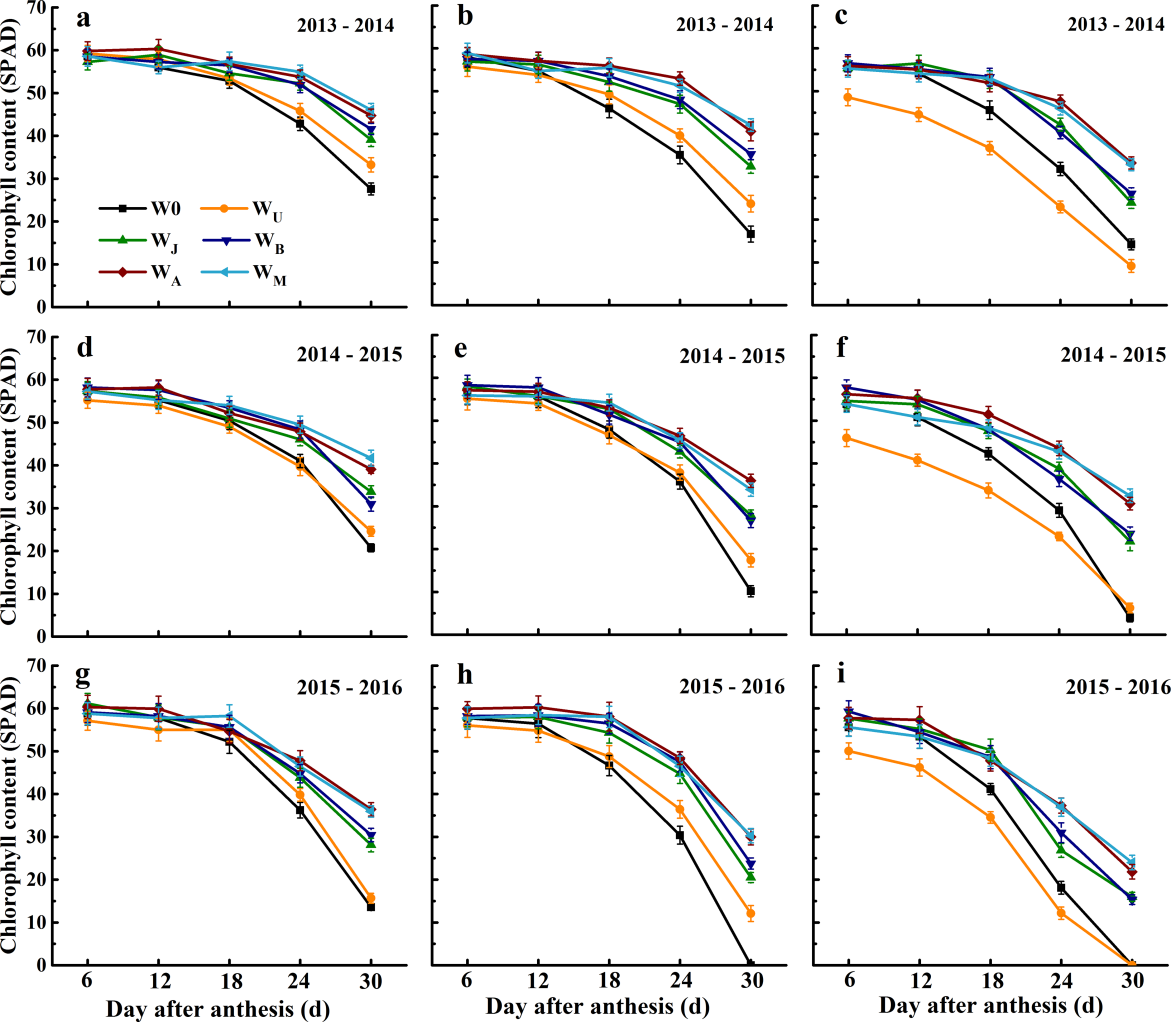
**Chlorophyll content (SPAD) of the flag leaf (a, d and g), second leaf (b, e and h), and third leaf (c, f and i) at 6, 12, 18, 24 and 30 day after anthesis under six treatments in the 2013–2016 growing season.** Vertical bars represent the standard errors. Mean values SE from three replicates.

The variations in chlorophyll content were consistent across three growing seasons (Fig 5). There was no significant difference in chlorophyll content of the flag leaf among all treatments from 6 to 18 days after anthesis (DAA; Fig 5A, 5D and 5G), and in the second leaf from 6 DAA to 12 DAA (Fig 5B, 5E and 5H). After 18 DAA (second leaf) or 24 DAA (flag leaf), the chlorophyll content in W0 and W_U_ treatments were significantly lower than they were in other treatments. At 30 DAA, the chlorophyll content of flag leaf and the second leaf in W_J_ and W_B_ were significantly lower than they were in W_A_ and W_M_ (Fig 5A, 5B, 5D, 5E, 5G and 5H). Compared with the other irrigation treatments, the chlorophyll content was lower in the third leaf under W_U_ treatments when measured from 6 DAA to 30 DAA (Fig 5C, 5F and 5I). There was no significant difference in the third leaf chlorophyll content among W_J_, W_B_, W_A_, W_M_ and W0 from 6 DAA to 12 DAA, whereas it decreased under W0 compared with W_J_, W_B_, W_A_ and W_M_ after 12 DAA. The same reduction was also observed in the third leaf under W_J_ and W_B_ compared with W_A_ and W_M_ after 18 DAA. Results showed that delayed irrigation slows down leaf senescence, which is beneficial for biomass accumulation after anthesis.

#### Source supply capacity

**Fig 6:**
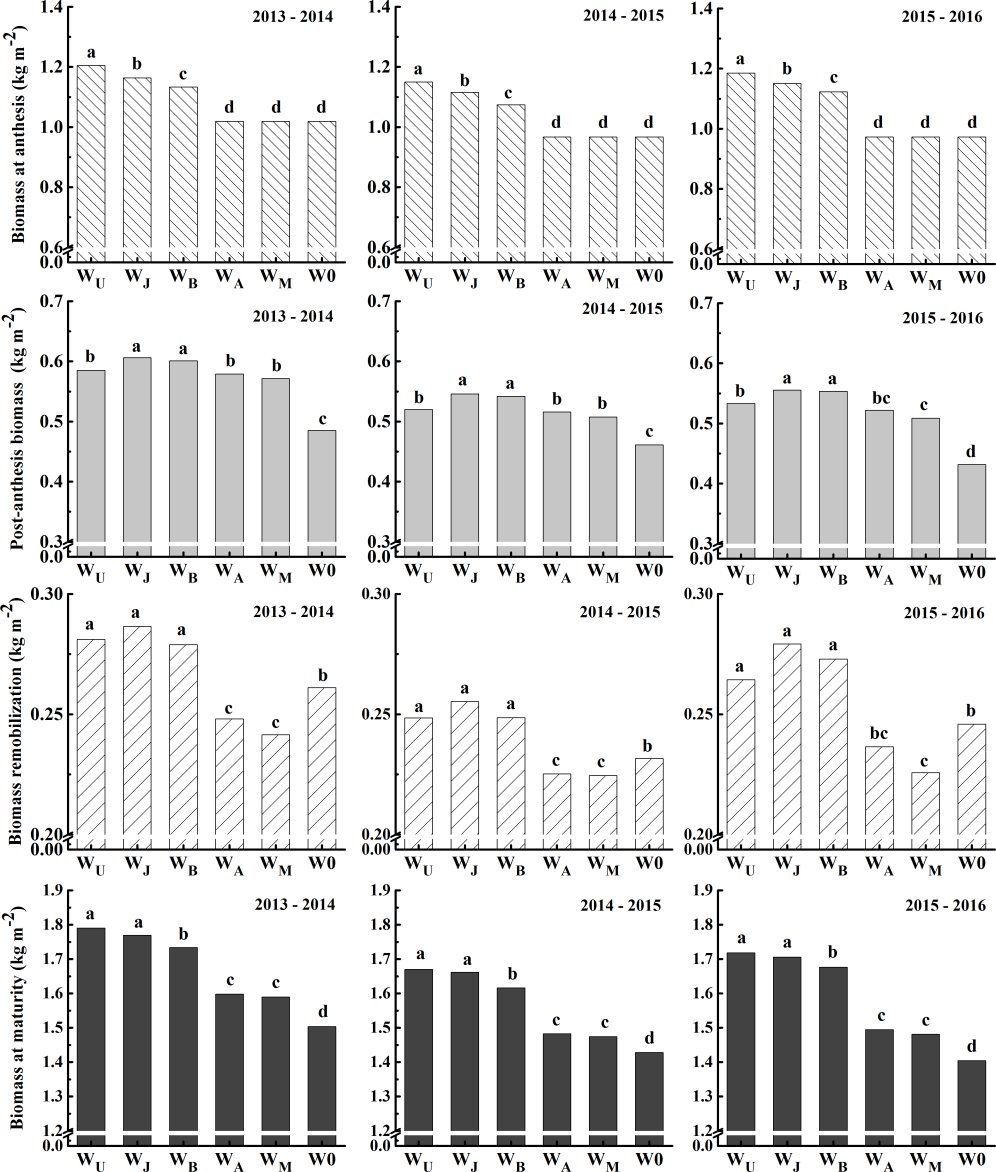
Biomass at anthesis and maturity, post-anthesis biomass and biomass remobilization under six treatments in the 2013–2014, 2014–2015 and 2015–2016 growing seasons. Different letters in the figure indicate statistical differences among treatments (LSD_*P*<0.05_).

Compared with W0, biomass at anthesis and maturity, and post-anthesis biomass from single irrigation treatments were higher in the 2013 to 2016 growing seasons (Fig 6). Under irrigation treatments, biomass at anthesis and maturity in W_J_ were higher than W_B_ and W_A_ and W_M_, but biomass at anthesis in W_J_ was lower than in W_U_, whereas no significant difference in biomass at maturity between W_J_ and W_U_ was identified in three growing seasons. The post-anthesis biomass in W_J_ was higher than in W_U_, W_A_ and W_M_, and there was no significant difference between W_J_ and W_B_, among W_U_, W_A_ and W_M_ in the 2013–2015 growing seasons and between W_U_ and W_A_ in the 2015–2016 growing season. The variations in post-anthesis biomass per unit anthesis leaf area (P_ost_BA-leaf ratio) were consistent across three growing seasons (Table 6). P_ost_BA-leaf ratio in W_J_ was significantly higher than W_U_, but lower than W_B_, W_A_, W_M_ and W0.

**Table 6:** P_ost_BA-leaf ratio, grain production efficiency (GPE), spike partitioning index (SPI) and harvest index (HI) in the 2013–2014, 2014–2015 and 2015–2016 growing seasons.

Treatments	P_ost_BA-leaf ratio ^1^	GPE	SPI	HI
	(g m^-2^)	(grains g^-1^)		
2013–2014				
W_U_	95.8d ^2^	17.9bc	0.168c	0.484d
W_J_	131.0c	19.6a	0.182a	0.505bc
W_B_	157.7b	18.4b	0.180a	0.507b
W_A_	179.3a	18.3b	0.174b	0.518a
W_M_	176.9a	17.8bc	0.174b	0.511ab
W0	150.3b	17.6c	0.174b	0.496c
2014–2015				
W_U_	82.5d	19.1c	0.169b	0.460d
W_J_	111.5c	20.9a	0.183a	0.482c
W_B_	129.8ab	20.0b	0.182a	0.489b
W_A_	137.8a	20.0b	0.179a	0.500a
W_M_	135.5a	19.1c	0.179a	0.496a
W0	123.2b	18.9c	0.179a	0.485bc
2015–2016				
W_U_	83.2d	18.3c	0.167c	0.464d
W_J_	114.4c	19.7a	0.186a	0.489bc
W_B_	135.9b	19.0b	0.185a	0.493b
W_A_	154.6a	19.1b	0.177b	0.507a
W_M_	150.8a	18.3c	0.177b	0.496b
W0	128.0bc	18.1c	0.177b	0.483c

^1^ P_ost_BA-leaf ratio, post-anthesis biomass per unit anthesis leaf area.

^2^ Mean values within columns followed by the different letters are statistically significant at *P* < 0.05 level.

Biomass remobilization in W_J_ was highest; there was no significant difference among W_U_, W_J_ and W_B_ in three growing seasons, whereas they were higher than in the rest of the treatments. It indicated that single irrigation at jointing could improve post-anthesis biomass and biomass remobilization, which was beneficial for improving grain yield.

### Sink capacity, grain production efficiency (GPE), spike partitioning index (SPI) and harvest index (HI)

**Fig 7:**
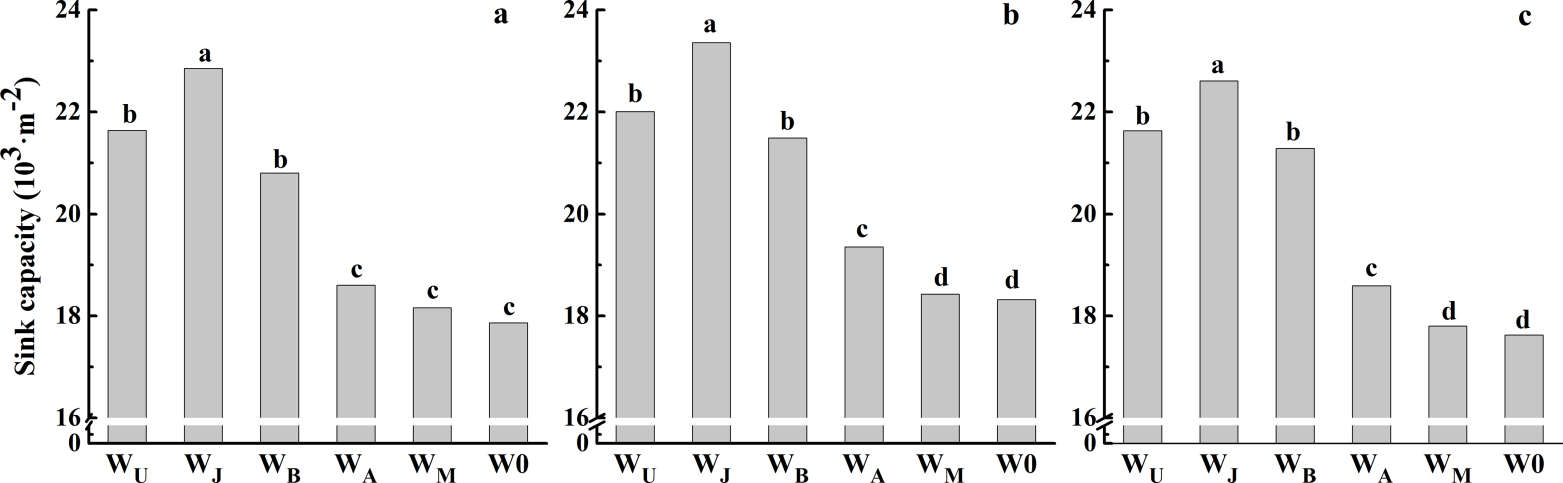
**Sink capacity under six treatments in the 2013–2014 (a), 2014–2015(b) and 2015–2016 (c) growing seasons.** Different letters in the figure indicate statistical differences among treatments (LSD_*P*<0.05_).

Compared with W0, irrigation treatments significantly increased sink capacity. The highest sink capacity was obtained in W_J_, and followed by W_U_, W_B_, W_A_, and W_M_ in the 2013 to 2016 growing seasons (Fig 7). As shown in Table 6, the highest GPE was obtained in W_J_, exceeding the mean values recorded in W_U_, W_B_, W_A_, W_M_ and W0 by 8.9%, 4.9%, 4.9%, 9.1% and 10.3%, respectively. The SPI was highest in W_J_, however, there was no significant difference between W_J_ and W_B_ in the 2013–2014 and the 2015–2016 growing seasons, or among W_J_, W_B_, W_A_, W_M_ and W0 in the 2014–2015 growing season. The highest HI was obtained in W_A_, while the lowest one was obtained in W_U_, and no significant difference was obtained among W_J_, W_B_ and W_M_ in the 2013–2014 and 2015–2016 growing seasons. These results showed that irrigation at jointing could obtain the highest sink capacity, GPE and SPI, compared to other treatments.

## Discussion

Our results showed that under conditions of adequate soil moisture before sowing, single irrigation treatments significantly improved grain yield compared to no irrigation treatment after sowing (W0), indicating that winter wheat with supplemental irrigation could lead to improved grain yield compared to rain fed [7, 29]. The grain yield and WUE of single irrigation treatments varied from 7380.4 to 9187.7 kg ha^-1^ and from 17.7 to 21.8 kg ha^-1^ mm^-1^ in three-year experiment, respectively, and that irrigation treatment at jointing (W_J_) obtained the highest grain yield (8217.7–9187.7 kg ha^-1^) and WUE (19.2–21.8 kg ha^-1^ mm^-1^). Irrigation treatment at booting (W_B_) observed the same level of grain yield and WUE as W_J_ (Table 5). It indicated that single irrigation from jointing to booting could obtain the highest grain yield and WUE.

Reducing irrigation frequency led to the reduced ET, decreased water irrigation amount, and increased WUE [7–8]. Interestingly, soil water storage consumption presented a negative correlation with irrigation frequency and irrigation amount [6, 30]. It was reported that, compared with two or three irrigation schemes, single irrigation decreased the ET, increased WUE and soil water storage consumption in the soil layers below 140 cm [6, 31]. Single irrigation at different growth stages also had an impact on ET [32–33]. In this study, the ET was decreased from 430.8–452.0 mm to 389.4–417.8 mm when single irrigation was delayed from upstanding to medium milk stage; early irrigation (W_U_) increased the ET pre-anthesis, while delayed irrigation increased ET post-anthesis (Table 4). Compared with W_U_, W_J_ and W_B_ reduced top three leaf size and population LAI, so reduced transpiration and water consumption pre-anthesis, which was consistent with the findings of Izanloo et al [34]. Compared with W_J_ and W_B_, W_U_ decreased the post-anthesis ET, this was because W_U_ over-consumed soil water storage above 120 cm soil layers pre-anthesis, and decreased available soil water storage post-anthesis (Fig 2). However, W_J_ and W_B_ maintained the higher soil water content in the 0 to 180 cm soil layers post-anthesis, delayed leaf senescence, and then increased physical water consumption demand [35]; therefore, W_J_ and W_B_ increased post-anthesis ET in comparison to W_U_. Grain yield was strongly influenced by the pattern of water used during the growing season and emphasized the importance of adequate water supply after anthesis for higher yield and WUE [36]. In this present study, W_J_ and W_B_ balanced pre- and post-anthesis water consumption and ensured post-anthesis water supply (Table 4 and Fig 2), and it was beneficial to improve grain yield and WUE.

Improving sink and source capacity simultaneously, and coordinating the “sink-source” relationships is a highly promising approach to increase biomass and yield [8, 13, 16]. Irrigation event can affect source and sink capacity and further influence grain yield [16, 18, 37]. Theoretically, increasing leaf area and maintaining leaf activity after anthesis is more important for dry matter production and grain yield [38]. In this research, the earlier irrigation, the larger scale in the top three leaves area and LAI (Figs 3 and 4), in contrast with previous research studies [8, 11, 29]. W_U_ got the highest top three leaves area and LAI, which resulted in highest biomass at anthesis and maturity (Figs 3, 4 and 6). W_J_ and W_B_ decreased the LAI at anthesis, but W_J_ and W_B_ obtained the higher post-anthesis biomass and HI than W_U_, because W_J_ and W_B_ maintained higher chlorophyll content in the top two leaves after 24 DAA, in the third leaf after anthesis, and improved P_ost_BA-leaf ratio (Figs 4, 5 and 6, Table 6). Additionally, W_J_ and W_B_ extended the duration of grain filling with improved leaf structure and viability, hence improved post-anthesis biomass (Table 3, Fig 4). However, smaller populations of W_A_ and W_M_ limited increase of post-anthesis biomass [39]. Previous studies demonstrated that biomass remobilization has a crucial impact on grain yield and is affected by soil water condition post-anthesis [29, 31, 40]. Compared with two or three irrigation schemes, single irrigation could increase biomass remobilization to ensure the stability of grain yield [10, 31]. In the current study, W_J_ obtained the highest biomass remobilization (Fig 6), findings that were consistent with previous studies [29]; however, we found there was no significant difference in biomass remobilization among W_J_, W_U_ and W_B_ (Fig 6). Compared with W0, W_A_ and W_M_ were conductive to a larger supply of assimilates for grain filling, thus reducing the need for biomass remobilization [41]. These results indicated that population source supply capacity was higher when single irrigation was applied at jointing and booting than in other treatments.

Increasing grain number per unit area (sink capacity) was an avenue to increase yield potential [20, 38]. Sink capacity was determined during the stem elongation period and around anthesis by soil water status [17, 39, 42]. Bindraban et al. [43] described that sink capacity is the result of biomass at anthesis and grain production efficiency (GPE). Previous studies have also shown that enhanced sink capacity can be achieved by increasing spike dry matter or SPI and GPE [44–48]. In the present research, compared with W_U_, W_J_ reduced leaf size and LAI, and thus decreased biomass at anthesis, but W_J_ improved the allocation of biomass to spike at anthesis, manifested by a higher SPI and GPE, subsequently increasing sink capacity (Table 6). Compared with W0, W_A_ and W_M_, W_J_ increased biomass at anthesis, SPI and GPE; therefore, W_J_ also obtained higher sink capacity than W0, W_A_ and W_M_ (Figs 6 and 7, Table 6). In summary, single irrigation at jointing or between jointing and booting improved sink capacity and source supply capacity simultaneously, coordinated the “sink-source” relationships, and thus improved grain yield and WUE.

## Conclusions

Under conditions of adequate soil moisture (80% of field capacity) before sowing, single irrigation applied at jointing (W_J_) or between jointing and booting (W_B_) with 75mm of irrigation was found to be the optimal irrigation scheme for high grain yield and WUE of winter wheat in NCP. The following points can be summarized: firstly, compared with irrigation at upstanding, W_J_ and W_B_ reduced pre-anthesis soil water storage consumption and total ET, maintaining higher soil water content above 180 cm soil layers for wheat growth after anthesis; secondly, W_J_ and W_B_ established optimized population and individual plant leaf size, delayed leaf senescence rate, extended longer grain-filling duration, improved P_ost_BA-leaf ratio and post-anthesis biomass, also increased biomass remobilization (source supply capacity), compared with W_U_; thirdly, compared with other treatments, W_J_ and W_B_ optimized the allocation of assimilation at anthesis, increased the spike partitioning index, maintained high grain production efficiency, and then achieved high sink capacity. W_A_ and W_M_ maintained high post-anthesis biomass per unit anthesis leaf area with slower leaf senescence rate, and induced low total ET; however, sink and source supply capacity, grain yield and WUE in W_A_ and W_M_ were lower than in W_J_. In summary, compared with other treatments, W_J_ and W_B_ improved source supply capacity and W_J_ improved sink capacity; W_B_ also improved sink capacity in comparison to W0, W_A_ and W_M_. W_J_ and W_B_ coordinated the “sink-source” relationships, and ultimately increased grain yield and WUE of winter wheat.

## Supporting information

S1 Dataset (PDF)
