# Supplementary material for: Optimizing single irrigation scheme to improve water use efficiency by manipulating winter wheat sink-source relationships in Northern China Plain
Source: PLoS One. 2018 Mar 8;13(3):e0193895. doi: 10.1371/journal.pone.0193895 (PMC5843274; doi:10.1371/journal.pone.0193895)
Supplement: S1 Dataset — (PDF) [file pone.0193895.s001.pdf]

The daily mean air temperature (℃) data corresponding to Fig 1

Date	Year			Date	Year			Date	Year			Date	Year			Date	Year			Date	Year			Date	Year			Date	Year			Date	Year		
	2013	2014	2015		2013	2014	2015		2013	2014	2015		2014	2015	2016		2014	2015	2016		2014	2015	2016		2014	2015	2016		2014	2015	2016		2014	2015	2016
1/10	.	.	.	1/11	8.5	11.6	10.5	1/12	2.2	-2.7	0.1	1/1	2.7	-5.4	1.9	1/2	0.7	-3.1	-5.6	1/3	4.4	4	3.8	1/4	17	6.1	18.8	1/5	21.5	21.6	23.7	1/6	24.4	26.8	21.2
2/10	.	.	.	2/11	9.3	8.3	10.3	2/12	2.2	-3.6	1.1	2/1	3.2	-3.3	1.1	2/2	2.7	-1.4	-3.7	2/3	4.4	5.7	8.9	2/4	15.4	5	14.6	2/5	17.5	17.9	15.7	2/6	19.9	26.7	22.8
3/10	.	.	.	3/11	10.8	8.9	11.5	3/12	3	-2.4	0.6	3/1	1.8	-0.6	0.7	3/2	-0.4	-1.9	-2.6	3/3	6.5	4.7	13.8	3/4	10.3	8.1	13.8	3/5	18.1	17.5	16.9	3/6	23.3	24.5	23.6
4/10	.	.	.	4/11	8.9	10.2	12.8	4/12	4.1	-3.4	-0.9	4/1	-0.5	2.2	0.1	4/2	-3.8	-1.1	0.8	4/3	5.6	1	11.2	4/4	11.8	9.9	12.9	4/5	14.7	16.8	19.9	4/6	24.1	20.8	23.9
5/10	.	.	.	5/11	11.7	10.5	9	5/12	4	-4.5	1	5/1	-1.5	2.6	-1.3	5/2	-1.3	-1.7	-1.1	5/3	2.9	2.7	7.9	5/4	9.6	11.2	14.3	5/5	14.5	19.3	22.4	5/6	26.2	23.6	23.5
6/10	.	.	.	6/11	13.3	8.9	2.7	6/12	1.6	-2	1.3	6/1	-3.2	-2.2	-1.5	6/2	-1.6	0.3	-3.6	6/3	2.2	3.9	9.7	6/4	13.7	6	16.9	6/5	15.1	16.4	18.5	6/6	25	25.4	24
7/10	.	.	.	7/11	8.3	6.3	3.3	7/12	1.2	-1.5	2.6	7/1	-0.5	-2.2	-3.7	7/2	-1.7	-0.7	-2	7/3	1.9	7.3	8	7/4	14.4	5.5	18.5	7/5	18.7	16.7	19.8	7/6	22.9	23.2	22.4
8/10	.	.	.	8/11	10.4	7.4	4.2	8/12	2.4	-2.6	3.6	8/1	-2.7	-2.7	-5	8/2	-3.2	-4.8	2.7	8/3	6	5.3	2.1	8/4	16.7	7.7	16.1	8/5	18.4	18.8	17.4	8/6	25.6	24	24.4
9/10	.	.	.	9/11	10.6	6.1	5.7	9/12	1.3	-1.2	4.6	9/1	-6.2	-1.4	-3.5	9/2	-5.2	-1.5	4.2	9/3	6.2	1.2	0.3	9/4	18.8	10	17.4	9/5	20.9	13.1	15.5	9/6	24.2	26.6	26.9
10/10	.	.	.	10/11	4.4	8.6	5.7	10/12	0.9	-1.2	3.4	10/1	-4.8	2.9	-2.3	10/2	-6	3.1	4.9	10/3	4.5	1.2	0.9	10/4	12.4	12.5	11.8	10/5	20.9	10.7	18.7	10/6	24.2	25.3	28.5
11/10	16.6	17.7	14.6	11/11	4.2	9.9	6.4	11/12	0.2	-2.8	1.2	11/1	-3.2	1.4	-5.3	11/2	-5.3	-0.1	4	11/3	10.9	3.6	3	11/4	10.5	12.9	9.9	11/5	13.6	13	22.4	11/6	22.1	22.4	25.4
12/10	20.6	12.5	15.2	12/11	5.9	4.3	7.9	12/12	-2.2	-2.7	0.3	12/1	-3.6	-2.2	-7.3	12/2	-3.3	-0.2	6.2	12/3	5.7	6.1	4.1	12/4	10.7	8.8	13	12/5	19.7	19	16	12/6	25.8	24	27.4
13/10	17.2	11.2	16.5	13/11	5.6	3.1	7.9	13/12	3.3	-1.1	0.7	13/1	-4.4	-0.6	-3.8	13/2	-0.6	1.7	0.1	13/3	6	5.6	4.7	13/4	13.7	7.4	15.7	13/5	22	21.3	16.4	13/6	27.5	22.9	25.9
14/10	12.2	12.1	18.8	14/11	6.3	4.7	9.1	14/12	-2.3	-2.2	2.5	14/1	-4.1	-0.7	-3.5	14/2	-0.7	6.7	-3.9	14/3	8.6	7.2	5.5	14/4	17.7	12.3	19	14/5	18.5	20.6	13	14/6	29	24.5	21
15/10	8.7	15.2	17.9	15/11	7.9	5.5	9.8	15/12	-1.8	-1	0.4	15/1	-2.5	-0.8	-0.9	15/2	2	3.3	-1.8	15/3	10.6	10.4	9.4	15/4	15.3	18.3	17.9	15/5	20.4	20	16.1	15/6	25.7	24.8	23.3
16/10	9.5	14	16.2	16/11	8	5.5	7.6	16/12	-2.6	-2.9	-0.9	16/1	-1.7	-0.7	-3.9	16/2	2	4.3	1.8	16/3	12	13.2	8.6	16/4	15.7	13.5	14.3	16/5	21.4	20.7	19.9	16/6	24.4	27	26.8
17/10	10.7	17.1	18.8	17/11	3.8	3.5	5.9	17/12	-0.5	-1.7	-3.3	17/1	-2.5	-3	-4.2	17/2	1.2	4.2	3.4	17/3	13	8.9	12.8	17/4	13.2	15.9	13	17/5	21.5	22.5	21.7	17/6	24.6	27.4	29.9
18/10	12.2	18.5	16.4	18/11	2.1	4.3	4.5	18/12	-5.4	0.9	-1.9	18/1	-4	3.5	-8.3	18/2	-0.3	1.3	2.9	18/3	10.1	7.5	13.1	18/4	12.9	15.2	15	18/5	22.1	23.2	22.1	18/6	24.3	25.9	28.1
19/10	12.6	15.7	14.4	19/11	1.1	5.5	5.7	19/12	-5.3	0.8	-0.7	19/1	-0.1	1.3	-7.9	19/2	-1	1	3.5	19/3	8.3	8.9	12.1	19/4	14.1	9.8	15.3	19/5	22.5	21.6	21.5	19/6	22.3	21.7	29.7
20/10	10.1	15.5	16.3	20/11	1.9	7.9	7.2	20/12	-5.6	-4.4	0.3	20/1	-2.2	1	-3.7	20/2	-0.6	3.7	0.9	20/3	8.9	11.3	10.4	20/4	14.1	11.5	16.2	20/5	23.8	21	20.4	20/6	20.8	23.2	28
21/10	10.7	13.9	13.6	21/11	3.1	6.3	5.1	21/12	-5.1	-4.5	0.1	21/1	-5.4	-0.5	-3.4	21/2	4.7	3	1.3	21/3	10.8	12	10.3	21/4	16.2	15.8	19.7	21/5	25.4	18.2	22	21/6	.	.	.
22/10	12.5	11.8	10.2	22/11	3.3	5.7	0	22/12	-4.4	-1.4	1.4	22/1	-1.6	-2.9	-9.2	22/2	3.9	-1.4	3.7	22/3	13.6	11.3	10	22/4	17.7	16.8	17.7	22/5	26.3	19.8	20.8	22/6	.	.	.
23/10	10.6	14.4	10.3	23/11	4.7	7.6	-2.6	23/12	-3.8	0.3	-2.2	23/1	0.4	0.4	-13.9	23/2	2.7	-0.3	0.2	23/3	14.1	8.2	8.4	23/4	18	19.8	18.8	23/5	26.7	22.5	20	23/6	.	.	.
24/10	8	18.3	10.5	24/11	5.4	4.8	-4.6	24/12	-4	-1	-1.8	24/1	1.5	-0.2	-12.1	24/2	4.7	1.7	0.6	24/3	14.9	10.9	9	24/4	19.3	19.1	18.3	24/5	22.7	24.1	19.9	24/6	.	.	.
25/10	8.3	21.5	12.6	25/11	3.2	4.4	-5	25/12	-4.1	-2.9	-1.3	25/1	0.7	-0.7	-5.8	25/2	8.6	2.9	2.9	25/3	15.4	11.5	9.1	25/4	20.6	21.4	19.6	25/5	23.9	23.9	19.3	25/6	.	.	.
26/10	8.2	15.1	13.4	26/11	1.8	5	-8.6	26/12	-5.2	-1.7	-1.4	26/1	-0.7	-0.2	-5.1	26/2	8.5	1.1	3	26/3	15.6	11.5	8.7	26/4	13.3	21.9	18.6	26/5	25.8	23.6	21.9	26/6	.	.	.
27/10	10.3	9.9	10.7	27/11	-1.8	5.8	-6.4	27/12	-5.6	-0.3	-3.6	27/1	1.9	-3.6	-4	27/2	6.2	0.7	4.2	27/3	18.7	13.6	12.2	27/4	15.9	21.3	15.9	27/5	26.4	23.8	21.1	27/6	.	.	.
28/10	11.9	9.7	10.4	28/11	-1.8	4.3	-2.2	28/12	-5.1	-1.3	-3.1	28/1	-0.1	-4	-1.5	28/2	3.8	0.1	2.6	28/3	17.3	15.3	13.4	28/4	15.9	17.5	18.6	28/5	26	23.5	20.4	28/6	.	.	.
29/10	11.6	11.7	7.9	29/11	2.4	3.2	-1.1	29/12	-2.6	0.3	-1.1	29/1	2.7	-1.7	-2.4	29/2	.	.	.	29/3	14	17.7	13.6	29/4	17.6	16.9	19.4	29/5	28.8	21.6	22.1	29/6	.	.	.
30/10	8.7	11	6.7	30/11	2.2	4.5	-2.6	30/12	-0.9	0.4	-1.3	30/1	1.6	-4	-4.8	30/2	.	.	.	30/3	16.1	17.6	15.7	30/4	19.4	21.5	23.2	30/5	29.3	23.2	24.8	30/6	.	.	.
31/10	8	12.9	7.5	.	.	.	.	31/12	2	-2.9	-0.9	31/1	-0.8	-4.4	-5.8	31/2	.	.	.	31/3	18	9.7	17.2	.	.	.	.	31/5	27.9	26.3	24	31/6	.	.	.

The precipitation (mm) data corresponding to Fig 1

Date	Precipitation	Date	Precipitation	Date	Precipitation
14 Oct. 2013	24.6	27 Nov. 2014	13.3	25 Oct. 2015	6.9
28 Oct. 2013	5.8	30 Nov. 2014	3.4	5 Nov. 2015	10.5
1 Nov. 2013	3.4	1 Jan. 2015	3.4	6 Nov. 2015	9.8
9 Nov. 2013	6.3	15 Feb. 2015	11.6	13 Nov. 2015	3.6
27 Feb. 2014	8.5	31 Mar. 2015	7.5	22 Nov. 2015	27.1
17 Apr. 2014	10.1	1 Apr.2015	22.0	23 Nov. 2015	3.1
25 Apr. 2014	8.6	8 Apr. 2015	3.0	8 Jan. 2016	8.0
11 May 2014	39.2	11 Apr. 2015	6.5	13 Feb. 2016	30.6
24 May 2014	12.0	18 Apr. 2015	6.3	16 Apr. 2016	5.5
1 Jun. 2014	3.4	27 Apr. 2015	9.8	17 Apr. 2016	5.3
4 Jun. 2014	5.4	2 May 2015	12.0	2 May 2016	9.7
.	.	9 May 2015	24.2	14 May 2016	14.3
.	.	11 May 2015	5.0	.	.
.	.	5 Jun. 2015	18.0	.	.
.	.	7 Jun. 2015	22.5	.	.
Total	127.3	Total	168.5	Total	134.4

The soil water content (%) data corresponding to Fig 2

Soil Layers(cm)	W0				W_U				W_J				W_B				W_A				W_M			
	J[1]	A	MM	M	J	A	MM	M	J	A	MM	M	J	A	MM	M	J	A	MM	M	J	A	MM	M
	2013-2014																							
20	11.44	8.08	7.91	7.84	19.14	8.60	8.20	8.22	11.44	10.61	8.01	7.94	11.44	11.14	8.30	7.61	11.44	8.08	11.01	8.28	11.44	8.08	7.91	11.14
40	12.18	7.93	7.52	7.73	18.06	7.94	8.60	8.13	12.18	10.75	8.82	8.39	12.18	12.24	9.56	8.70	12.18	7.93	11.21	9.02	12.18	7.93	7.52	11.58
60	14.08	9.29	8.32	8.19	17.92	10.08	9.01	8.48	14.08	11.58	9.57	8.58	14.08	12.32	10.08	9.03	14.08	9.29	11.58	9.48	14.08	9.29	8.32	11.07
80	17.96	11.80	9.81	9.31	18.49	12.66	10.28	9.50	17.96	14.62	12.10	10.36	17.96	14.15	11.96	10.27	17.96	11.80	12.19	9.89	17.96	11.80	9.81	10.47
100	18.83	13.57	11.66	10.69	19.34	15.90	12.72	11.38	18.83	16.22	13.84	11.70	18.83	16.30	14.45	12.22	18.83	13.57	13.82	12.57	18.83	13.57	11.66	11.10
120	21.49	16.44	14.78	12.99	21.50	19.32	16.29	14.06	21.49	18.80	17.01	14.47	21.49	19.36	17.88	14.96	21.49	16.44	16.60	15.49	21.49	16.44	14.78	14.07
140	22.59	19.47	17.49	15.45	22.21	20.82	18.24	16.34	22.59	20.43	19.25	16.98	22.59	20.97	19.81	17.15	22.59	19.47	19.02	17.99	22.59	19.47	17.49	16.85
160	22.95	21.90	19.74	18.55	23.11	22.27	20.47	18.87	22.95	22.94	21.72	19.48	22.95	22.40	21.56	19.95	22.95	21.90	21.52	20.56	22.95	21.90	19.74	19.03
180	23.67	23.09	22.55	21.26	23.66	23.11	22.81	22.08	23.67	23.41	23.09	22.22	23.67	23.90	23.41	22.75	23.67	23.09	22.96	22.42	23.67	23.09	22.55	22.24
200	23.85	23.73	23.52	22.95	23.94	23.88	23.93	23.17	23.85	23.57	24.06	23.28	23.85	24.13	23.91	23.59	23.85	23.73	23.80	23.39	23.85	23.73	23.52	23.18
	2014-2015																							
20	15.75	11.05	9.23	14.03	20.99	14.87	10.84	13.83	15.75	14.63	11.35	13.65	15.75	15.42	11.52	14.26	15.75	11.05	14.98	15.33	15.75	11.05	9.23	16.24
40	13.75	10.08	8.82	7.77	20.11	13.57	9.68	9.30	13.75	14.80	11.95	9.46	13.75	15.19	11.82	9.33	13.75	10.08	14.30	9.46	13.75	10.08	8.82	12.88
60	15.60	12.26	9.59	8.49	19.83	13.08	10.25	9.36	15.60	14.51	12.12	9.17	15.60	15.01	12.08	9.44	15.60	12.26	12.49	9.65	15.60	12.26	9.59	10.67
80	18.45	13.52	12.07	10.21	19.31	14.00	12.09	10.78	18.45	15.79	13.16	11.07	18.45	15.95	13.31	11.08	18.45	13.52	12.88	10.42	18.45	13.52	12.07	11.45
100	20.82	15.90	13.25	11.24	20.99	16.67	14.51	12.11	20.82	18.66	14.50	12.19	20.82	18.02	15.13	12.61	20.82	15.90	14.79	12.32	20.82	15.90	13.25	12.63
120	21.37	18.01	15.15	12.68	21.21	18.05	16.16	13.35	21.37	19.42	16.86	14.12	21.37	20.06	17.73	14.23	21.37	18.01	17.04	14.70	21.37	18.01	15.15	14.57
140	21.71	19.70	17.07	15.09	21.80	19.86	18.45	15.27	21.71	20.35	19.46	17.43	21.71	21.71	19.82	16.95	21.71	19.70	18.08	17.25	21.71	19.70	17.07	16.47
160	21.99	20.63	18.46	16.16	22.47	21.11	19.99	17.31	21.99	21.75	20.92	18.89	21.99	21.52	21.34	18.89	21.99	20.63	19.00	18.22	21.99	20.63	18.46	17.74
180	22.18	22.17	21.29	19.81	23.08	22.86	22.13	21.65	22.18	22.34	23.26	21.33	22.18	22.55	23.11	21.62	22.18	22.17	22.56	22.05	22.18	22.17	21.29	21.03
200	23.65	23.69	23.85	22.89	22.92	22.95	23.05	22.82	23.65	23.76	23.73	23.31	23.65	23.08	23.97	23.24	23.65	23.69	24.62	24.24	23.65	23.69	23.85	22.97
	2015-2016																							
20	15.37	9.64	8.74	7.41	22.84	11.15	7.83	7.33	15.37	13.38	8.53	8.00	15.37	14.09	8.95	7.66	15.37	9.64	11.22	8.56	15.37	9.64	8.74	11.96
40	16.44	10.31	8.03	7.85	22.03	12.21	8.10	7.51	16.44	13.99	8.73	8.05	16.44	14.65	9.41	7.83	16.44	10.31	10.45	8.39	16.44	10.31	8.03	11.74
60	18.49	12.45	8.65	8.48	20.55	13.19	8.60	8.13	18.49	14.16	10.16	8.56	18.49	15.91	10.98	8.09	18.49	12.45	10.50	8.59	18.49	12.45	8.65	9.84
80	19.67	14.37	10.33	9.44	21.68	15.87	11.15	9.86	19.67	17.25	13.02	10.08	19.67	16.61	13.74	10.29	19.67	14.37	12.44	9.84	19.67	14.37	10.33	9.91
100	20.11	16.01	12.34	10.27	20.80	17.07	13.81	11.04	20.11	18.11	15.25	12.01	20.11	17.90	15.61	12.37	20.11	16.01	15.76	13.01	20.11	16.01	12.34	11.60
120	22.00	18.52	15.86	14.23	22.33	19.37	17.62	15.08	22.00	19.54	18.36	15.38	22.00	20.55	19.06	15.77	22.00	18.52	19.31	16.04	22.00	18.52	15.86	15.32
140	22.53	19.93	17.27	15.16	22.18	20.12	19.11	16.63	22.53	21.11	20.33	17.57	22.53	21.62	20.68	17.88	22.53	19.93	20.54	18.51	22.53	19.93	17.27	16.84
160	23.99	21.29	19.51	16.76	23.21	22.31	20.80	18.44	23.99	23.41	22.46	20.00	23.99	23.19	22.06	20.55	23.99	21.29	21.18	19.90	23.99	21.29	19.51	18.66
180	23.34	22.39	21.56	20.00	22.80	22.41	21.76	20.56	23.34	23.41	22.89	21.68	23.34	23.08	22.39	22.12	23.34	22.39	22.41	21.54	23.34	22.39	21.56	20.84
200	23.48	23.02	22.80	21.40	23.43	23.23	23.59	22.82	23.48	23.70	23.09	22.92	23.48	23.18	22.81	22.62	23.48	23.02	23.25	22.43	23.48	23.02	22.80	22.17

[1] J, Jointing stage; A, Anthesis stage; M, Medium milk stage; M, Maturity stage.

The leaf length (cm) data corresponding to Fig 3a, b and c

Treatments	Plant 1	Plant 2	Plant 3	Plant 4	Plant 5	Plant 6	Plant 7	Plant 8	Plant 9	Plant 10	Plant 11	Plant 12	Plant 13	Plant 14	Plant 15	Plant 16	Plant 17	Plant 18	Plant 19	Plant 20
	Flag leaf length																			
W_U	14.8	14.8	15.1	16.5	16.2	14.8	15.6	14.4	15.1	17.0	16.0	15.1	14.3	14.8	14.8	16.0	16.5	14.8	16.6	14.8
W_J	12.4	11.9	12.9	11.9	13.8	12.4	12.9	12.9	11.4	13.0	12.6	12.1	12.1	12.6	13.1	12.1	12.1	12.1	11.7	11.1
W_B	8.7	10.0	8.0	9.0	9.2	10.0	8.0	10.0	9.0	10.0	9.5	9.2	9.5	8.5	8.5	10.0	9.8	9.5	8.4	10.0
W_A	9.9	9.2	10.2	8.7	9.4	10.0	8.7	9.7	8.7	9.2	8.7	9.7	8.9	8.2	9.7	8.7	9.2	7.7	8.2	9.2
W_M	9.9	9.2	10.2	8.7	9.4	10.0	8.7	9.7	8.7	9.2	8.7	9.7	8.9	8.2	9.7	8.7	9.2	7.7	8.2	9.2
W0	9.9	9.2	10.2	8.7	9.4	10.0	8.7	9.7	8.7	9.2	8.7	9.7	8.9	8.2	9.7	8.7	9.2	7.7	8.2	9.2
	Second leaf length																			
W_U	21.5	21.9	19.9	20.5	21.4	21.7	22.3	21.0	20.3	19.8	18.6	18.6	18.7	19.7	21.0	21.5	21.8	19.5	20.9	21.9
W_J	15.2	14.7	15.2	16.2	15.3	16.3	17.6	16.0	16.7	16.0	18.0	16.6	17.0	17.7	16.5	16.0	15.8	16.7	16.0	15.2
W_B	14.0	14.3	14.3	14.7	15.0	14.2	14.2	14.0	14.7	14.1	15.4	13.9	14.9	15.3	13.8	14.9	15.2	15.3	14.9	13.7
W_A	13.7	13.7	14.7	14.8	14.1	14.8	14.3	14.7	14.0	14.4	15.3	13.9	14.4	14.9	15.2	15.0	15.6	14.6	15.4	13.7
W_M	13.7	13.7	14.7	14.8	14.1	14.8	14.3	14.7	14.0	14.4	15.3	13.9	14.4	14.9	15.2	15.0	15.6	14.6	15.4	13.7
W0	13.7	13.7	14.7	14.8	14.1	14.8	14.3	14.7	14.0	14.4	15.3	13.9	14.4	14.9	15.2	15.0	15.6	14.6	15.4	13.7
	Third leaf length																			
W_U	20.9	21.4	21.0	20.6	20.6	21.3	21.1	20.7	20.3	20.6	19.2	19.0	19.1	19.6	19.0	19.5	19.7	19.4	19.3	18.8
W_J	17.5	18.4	17.6	17.4	17.6	18.1	17.2	18.4	18.1	17.7	17.5	17.9	18.6	18.0	17.3	18.4	18.2	17.6	18.2	17.5
W_B	18.0	18.2	17.5	17.0	17.3	18.5	17.5	18.2	17.1	17.5	18.0	17.0	17.1	18.4	18.3	18.3	17.0	17.1	17.6	17.3
W_A	17.4	18.4	17.2	18.0	17.2	17.8	18.1	18.3	17.2	17.6	17.6	16.5	16.5	17.9	17.4	18.0	17.4	17.4	17.2	18.0
W_M	17.4	18.4	17.2	18.0	17.2	17.8	18.1	18.3	17.2	17.6	17.6	16.5	16.5	17.9	17.4	18.0	17.4	17.4	17.2	18.0
W0	17.4	18.4	17.2	18.0	17.2	17.8	18.1	18.3	17.2	17.6	17.6	16.5	16.5	17.9	17.4	18.0	17.4	17.4	17.2	18.0

The leaf width (cm) data corresponding to Fig 3d, e and f

Treatments	Plant 1	Plant 2	Plant 3	Plant 4	Plant 5	Plant 6	Plant 7	Plant 8	Plant 9	Plant 10	Plant 11	Plant 12	Plant 13	Plant 14	Plant 15	Plant 16	Plant 17	Plant 18	Plant 19	Plant 20
	Flag leaf width																			
W_U	1.6	1.6	1.6	1.8	1.7	1.6	1.7	1.6	1.7	1.8	1.7	1.6	1.6	1.6	1.6	1.7	1.8	1.7	1.8	1.6
W_J	1.5	1.5	1.5	1.4	1.5	1.4	1.5	1.5	1.5	1.4	1.5	1.5	1.5	1.4	1.5	1.5	1.4	1.5	1.5	1.4
W_B	1.3	1.3	1.3	1.4	1.3	1.5	1.5	1.3	1.3	1.4	1.3	1.4	1.4	1.3	1.3	1.4	1.4	1.3	1.3	1.3
W_A	1.4	1.3	1.5	1.3	1.3	1.4	1.5	1.4	1.4	1.4	1.3	1.3	1.2	1.4	1.4	1.4	1.3	1.2	1.2	1.2
W_M	1.4	1.3	1.5	1.3	1.3	1.4	1.5	1.4	1.4	1.4	1.3	1.3	1.2	1.4	1.4	1.4	1.3	1.2	1.2	1.2
W0	1.4	1.3	1.5	1.3	1.3	1.4	1.5	1.4	1.4	1.4	1.3	1.3	1.2	1.4	1.4	1.4	1.3	1.2	1.2	1.2
	Second leaf width																			
W_U	1.8	1.8	1.8	1.8	1.9	1.8	1.8	1.8	1.8	1.8	1.7	1.7	1.8	1.7	1.7	1.8	1.9	1.7	1.9	1.7
W_J	1.6	1.6	1.6	1.6	1.6	1.5	1.6	1.6	1.6	1.4	1.6	1.4	1.6	1.5	1.4	1.6	1.6	1.6	1.6	1.5
W_B	1.6	1.6	1.5	1.4	1.5	1.4	1.4	1.6	1.4	1.6	1.4	1.4	1.5	1.4	1.6	1.5	1.5	1.5	1.5	1.4
W_A	1.5	1.5	1.4	1.5	1.4	1.5	1.6	1.5	1.5	1.4	1.5	1.4	1.5	1.5	1.5	1.5	1.4	1.5	1.5	1.4
W_M	1.5	1.5	1.4	1.5	1.4	1.5	1.6	1.5	1.5	1.4	1.5	1.4	1.5	1.5	1.5	1.5	1.4	1.5	1.5	1.4
W0	1.5	1.5	1.4	1.5	1.4	1.5	1.6	1.5	1.5	1.4	1.5	1.4	1.5	1.5	1.5	1.5	1.4	1.5	1.5	1.4
	Third leaf width																			
W_U	1.3	1.4	1.4	1.4	1.3	1.2	1.3	1.2	1.4	1.2	1.3	1.2	1.3	1.2	1.2	1.2	1.4	1.3	1.2	1.2
W_J	1.2	1.3	1.1	1.1	1.1	1.2	1.2	1.2	1.3	1.2	1.1	1.1	1.2	1.3	1.1	1.3	1.1	1.1	1.2	1.2
W_B	1.2	1.2	1.2	1.1	1.1	1.2	1.1	1.2	1.1	1.1	1.2	1.2	1.1	1.1	1.2	1.1	1.1	1.1	1.1	1.1
W_A	1.1	1.3	1.1	1.1	1.1	1.1	1.3	1.1	1.2	1.2	1.1	1.1	1.1	1.1	1.3	1.1	1.1	1.1	1.1	1.2
W_M	1.1	1.3	1.1	1.1	1.1	1.1	1.3	1.1	1.2	1.2	1.1	1.1	1.1	1.1	1.3	1.1	1.1	1.1	1.1	1.2
W0	1.1	1.3	1.1	1.1	1.1	1.1	1.3	1.1	1.2	1.2	1.1	1.1	1.1	1.1	1.3	1.1	1.1	1.1	1.1	1.2

The leaf area (cm^2) data corresponding to Fig 3g, h and i

Treatments	Plant 1	Plant 2	Plant 3	Plant 4	Plant 5	Plant 6	Plant 7	Plant 8	Plant 9	Plant 10	Plant 11	Plant 12	Plant 13	Plant 14	Plant 15	Plant 16	Plant 17	Plant 18	Plant 19	Plant 20
	Flag leaf area																			
W_U	18.5	18.5	18.8	23.2	21.5	18.5	20.7	18.0	20.0	23.9	21.2	18.8	17.8	18.5	18.5	21.2	23.2	19.6	23.3	18.5
W_J	14.5	13.9	15.1	13.0	16.1	13.5	15.1	15.1	13.3	14.2	14.7	14.2	14.2	13.8	15.3	14.2	13.2	14.2	13.7	12.1
W_B	8.8	10.1	8.1	9.8	9.3	11.7	9.4	10.1	9.1	10.9	9.6	10.0	10.4	8.6	8.6	10.9	10.7	9.6	8.5	10.1
W_A	10.8	9.3	11.9	8.8	9.5	10.9	10.2	10.6	9.5	10.0	8.8	9.8	8.3	9.0	10.6	9.5	9.3	7.2	7.7	8.6
W_M	10.8	9.3	11.9	8.8	9.5	10.9	10.2	10.6	9.5	10.0	8.8	9.8	8.3	9.0	10.6	9.5	9.3	7.2	7.7	8.6
W0	10.8	9.3	11.9	8.8	9.5	10.9	10.2	10.6	9.5	10.0	8.8	9.8	8.3	9.0	10.6	9.5	9.3	7.2	7.7	8.6
	Second leaf area																			
W_U	30.2	30.7	27.9	28.8	31.7	30.5	31.3	29.5	28.5	27.8	24.7	24.7	26.3	26.1	27.8	30.2	32.3	25.9	31.0	29.0
W_J	19.0	18.3	19.0	20.2	19.1	19.1	22.0	20.0	20.8	17.5	22.5	18.1	21.2	20.7	18.0	20.0	19.7	20.8	20.0	17.8
W_B	17.5	17.8	16.7	16.1	17.6	15.5	15.5	17.5	16.1	17.6	16.8	15.2	17.4	16.7	17.2	17.4	17.8	17.9	17.4	15.0
W_A	16.0	16.0	16.1	17.3	15.4	17.3	17.8	17.2	16.4	15.7	17.9	15.2	16.8	17.4	17.8	17.6	17.0	17.1	18.0	15.0
W_M	16.0	16.0	16.1	17.3	15.4	17.3	17.8	17.2	16.4	15.7	17.9	15.2	16.8	17.4	17.8	17.6	17.0	17.1	18.0	15.0
W0	16.0	16.0	16.1	17.3	15.4	17.3	17.8	17.2	16.4	15.7	17.9	15.2	16.8	17.4	17.8	17.6	17.0	17.1	18.0	15.0
	Third leaf area																			
W_U	21.2	23.4	22.9	22.5	20.9	19.9	21.4	19.4	22.2	19.3	19.5	17.8	19.4	18.3	17.8	18.3	21.5	19.7	18.1	17.6
W_J	16.4	18.7	15.1	14.9	15.1	16.9	16.1	17.2	18.4	16.6	15.0	15.4	17.4	18.3	14.8	18.7	15.6	15.1	17.0	16.4
W_B	16.8	17.0	16.4	14.6	14.8	17.3	15.0	17.0	14.7	15.0	16.8	15.9	14.7	15.8	17.1	15.7	14.6	14.7	15.1	14.8
W_A	14.9	18.7	14.8	15.4	14.8	15.3	18.4	15.7	16.1	16.5	15.1	14.2	14.2	15.4	17.6	15.4	14.9	14.9	14.8	16.8
W_M	14.9	18.7	14.8	15.4	14.8	15.3	18.4	15.7	16.1	16.5	15.1	14.2	14.2	15.4	17.6	15.4	14.9	14.9	14.8	16.8
W0	14.9	18.7	14.8	15.4	14.8	15.3	18.4	15.7	16.1	16.5	15.1	14.2	14.2	15.4	17.6	15.4	14.9	14.9	14.8	16.8

The LAI data corresponding to Fig 4

Treatments	2013-2014		2014-2015		2015-2016	
	Leaf area index (LAI)					
	Top three leaves	Total green leaves	Top three leaves	Total green leaves	Top three leaves	Total green leaves
W_U	4.53	6.11	4.57	6.30	4.63	6.41
W_J	3.28	4.63	3.48	4.90	3.37	4.86
W_B	2.53	3.82	2.78	4.18	2.68	4.07
W_A	2.33	3.23	2.54	3.75	2.43	3.39
W_M	2.33	3.23	2.54	3.75	2.43	3.39
W0	2.33	3.23	2.54	3.75	2.43	3.39

The flag leaf SPAD data corresponding to Fig 5a, d and g

Date	Flag leaf SPAD											
	W0		W_U		W_J		W_B		W_A		W_M	
2013-2014	Mean	SE	Mean	SE	Mean	SE	Mean	SE	Mean	SE	Mean	SE
6DAA	58.6	2.28	59.3	1.90	57.3	1.85	58.5	1.63	59.8	2.19	58.6	2.28
12DAA	56.0	1.46	57.9	1.80	58.9	1.87	57.3	1.64	60.3	2.20	56.0	1.46
18DAA	52.8	1.73	53.4	1.55	54.6	1.61	56.5	1.61	56.8	1.62	57.4	2.17
24DAA	42.8	1.57	45.8	1.77	52.3	1.57	51.9	1.79	53.8	1.59	55.0	1.60
30DAA	27.6	1.40	33.2	1.66	39.1	1.55	41.6	1.29	44.7	1.50	46.1	1.52
2014-2015												
6DAA	57.3	2.01	55.2	1.88	57.4	2.19	58.2	2.21	57.8	2.54	57.3	1.85
12DAA	55.3	1.92	54.0	1.82	55.9	1.92	57.6	2.20	58.2	1.76	55.3	2.20
18DAA	50.4	2.05	49.1	1.55	50.9	2.00	53.5	1.65	52.2	1.62	54.0	2.12
24DAA	41.0	1.56	39.7	2.11	46.1	1.49	48.4	2.00	47.9	1.99	49.5	2.03
30DAA	20.8	0.93	24.6	1.17	33.8	1.39	31.0	1.66	39.1	0.94	41.7	1.87
2015-2016												
6DAA	58.8	2.69	57.1	2.22	61.1	2.39	59.1	2.38	60.3	2.71	58.8	2.57
12DAA	57.8	2.71	55.0	2.65	57.9	2.92	58.2	2.91	59.9	2.93	57.8	2.56
18DAA	52.2	2.71	55.0	2.68	55.7	2.75	55.7	2.75	54.7	2.73	58.2	2.62
24DAA	36.2	1.84	39.8	1.48	43.8	2.22	44.8	2.15	47.8	2.36	46.4	2.24
30DAA	13.6	0.66	15.7	1.15	28.1	1.59	30.4	1.59	36.4	1.57	35.9	1.31

The second leaf SPAD data corresponding to Fig 5b, e and h

Date	Second leaf SPAD											
	W0		W_U		W_I		W_B		W_A		W_M	
2013-2014	Mean	SE	Mean	SE	Mean	SE	Mean	SE	Mean	SE	Mean	SE
6DAA	57.9	2.30	55.7	2.28	56.9	2.17	57.5	1.61	58.7	1.51	58.9	2.30
12DAA	54.7	1.73	53.8	1.71	56.2	2.16	57.1	2.05	57.1	2.00	54.7	1.73
18DAA	46.0	2.17	49.3	2.20	52.1	2.13	53.5	1.74	55.9	1.84	55.4	2.15
24DAA	35.1	2.05	39.6	1.55	47.0	2.09	48.0	2.09	53.0	1.58	51.3	1.56
30DAA	16.6	1.87	23.7	1.94	32.4	1.56	35.3	1.26	40.6	2.23	42.1	1.48
2014-2015												
6DAA	55.9	2.16	55.1	2.65	58.0	1.71	58.3	2.32	57.1	1.95	55.9	1.97
12DAA	55.7	2.66	54.0	1.61	55.8	1.67	57.8	2.31	56.8	1.94	55.7	1.97
18DAA	47.9	2.02	46.6	2.02	52.8	1.63	51.4	2.18	53.0	1.88	54.2	1.96
24DAA	35.8	1.75	37.9	1.79	42.8	1.47	45.0	2.05	46.5	1.76	45.4	1.79
30DAA	10.2	1.29	17.4	1.57	27.9	1.23	26.7	1.63	36.0	1.54	33.9	1.55
2015-2016												
6DAA	57.8	2.61	56.0	2.80	57.9	2.17	58.2	1.94	59.9	1.65	57.8	2.59
12DAA	56.5	3.25	54.8	2.68	58.0	2.74	58.4	2.58	60.3	2.67	58.5	2.21
18DAA	46.7	2.36	48.8	2.53	54.3	2.37	56.5	2.39	58.1	3.33	58.0	2.50
24DAA	30.4	2.11	36.4	2.06	44.7	2.27	47.5	2.34	48.4	1.49	46.5	2.38
30DAA	0.0	0.00	12.1	1.87	20.5	1.17	23.8	1.31	30.0	1.87	30.3	1.67

The third leaf SPAD data corresponding to Fig 5c, f and i

Date	Third leaf SPAD											
	W0		W_U		W_I		W_B		W_A		W_M	
2013-2014	Mean	SE	Mean	SE	Mean	SE	Mean	SE	Mean	SE	Mean	SE
6DAA	55.3	1.99	48.6	1.97	55.3	1.63	56.5	2.00	55.9	2.16	55.3	1.99
12DAA	54.1	1.98	44.5	1.60	56.5	1.90	55.2	1.62	55.1	2.15	54.1	1.98
18DAA	45.6	2.16	36.7	1.53	52.7	2.06	53.3	2.06	51.9	2.05	53.0	1.52
24DAA	31.8	1.55	23.0	1.36	42.2	1.48	40.3	1.46	47.6	1.53	46.0	1.52
30DAA	14.2	1.28	9.2	1.51	23.9	1.33	26.1	1.34	33.2	1.41	32.8	1.41
2014-2015												
6DAA	53.9	1.66	46.0	2.00	54.6	1.89	57.8	1.75	56.2	1.93	53.9	1.93
12DAA	50.9	2.06	40.8	1.41	53.9	1.87	54.9	2.24	55.3	1.91	50.9	1.86
18DAA	42.3	1.42	33.7	1.75	47.6	1.77	48.0	1.57	51.5	1.87	48.3	1.85
24DAA	29.1	1.62	23.0	0.98	38.8	1.55	36.4	1.80	43.5	1.70	42.8	1.73
30DAA	4.0	1.00	6.2	1.13	21.8	2.15	23.6	1.59	30.6	1.49	32.5	1.58
2015-2016												
6DAA	55.6	2.04	50.1	1.90	57.7	1.56	59.3	2.51	57.7	2.52	55.6	2.14
12DAA	53.4	2.76	46.2	2.02	55.2	2.38	54.5	2.66	57.3	3.13	53.4	2.70
18DAA	41.2	1.30	34.5	1.33	50.3	2.55	48.6	2.71	47.8	2.45	48.4	1.92
24DAA	18.1	1.50	12.2	1.46	26.8	1.64	31.0	2.32	37.3	1.77	36.9	2.12
30DAA	0.0	0.00	0.0	0.00	16.0	1.07	15.3	1.15	21.8	1.69	24.0	1.70

The biomass accumulation and remobilization (kg m^{-2}) data corresponding to Fig 6

Treatments	Biomass accumulation			Biomass remobilization
	Anthesis	Maturity	Post-anthesis	
2013-2014				
W_U	1.205	1.790	0.585	0.281
W_J	1.163	1.769	0.606	0.287
W_B	1.133	1.734	0.601	0.279
W_A	1.019	1.598	0.579	0.248
W_M	1.019	1.590	0.571	0.241
W0	1.019	1.504	0.485	0.261
2014-2015				
W_U	1.150	1.670	0.520	0.248
W_J	1.116	1.662	0.546	0.255
W_B	1.074	1.616	0.542	0.249
W_A	0.967	1.483	0.516	0.225
W_M	0.967	1.475	0.507	0.224
W0	0.967	1.428	0.461	0.232
2015-2016				
W_U	1.185	1.718	0.533	0.264
W_J	1.151	1.706	0.555	0.279
W_B	1.123	1.677	0.553	0.273
W_A	0.973	1.495	0.522	0.237
W_M	0.973	1.482	0.509	0.226
W0	0.973	1.405	0.432	0.246

The sink capacity (10^3 m^{-2}) data corresponding to Fig 7

Treatments	Sink capacity		
	2013-2014	2014-2015	2015-2016
W_U	21.64	22.01	21.63
W_J	22.85	23.36	22.61
W_B	20.80	21.49	21.28
W_A	18.60	19.36	18.59
W_M	18.16	18.43	17.80
W0	17.86	18.32	17.63
